# The association between prescription drugs and colorectal cancer prognosis: a nationwide cohort study using a medication-wide association study

**DOI:** 10.1186/s12885-023-11105-9

**Published:** 2023-07-10

**Authors:** Hyeong-Taek Woo, Seung-Yong Jeong, Aesun Shin

**Affiliations:** 1 Department of Preventive Medicine, Keimyung University School of Medicine, 1095 Dalgubeol-daero, Dalseo- gu, Daegu, 42601 Korea; 2 Department of Preventive Medicine, Seoul National University College of Medicine, Seoul, Korea; 3 Department of Surgery, Seoul National University College of Medicine, Seoul, Korea; 4 Cancer Research Institute, Seoul National University, Seoul, Korea

**Keywords:** Colorectal cancer, Medication-wide association study, Pharmacovigilance, Hypothesis free, Agnostic, Korean population

## Abstract

**Background:**

With the availability of health insurance claim data, pharmacovigilance for various drugs has been suggested; however, it is necessary to establish an appropriate analysis method. To detect unintended drug effects and to generate new hypotheses, we conducted a hypothesis-free study to systematically examine the relationship between all prescription nonanticancer drugs and the mortality of colorectal cancer patients.

**Methods:**

We used the Korean National Health Insurance Service-National Sample Cohort database. A total of 2,618 colorectal cancer patients diagnosed between 2004 and 2015 were divided into drug discovery and drug validation sets (1:1) through random sampling. Drugs were classified using the Anatomical Therapeutic Chemical (ATC) classification system: 76 drugs classified as ATC level 2 and 332 drugs classified as ATC level 4 were included in the analysis. We used a Cox proportional hazard model adjusted for sex, age, colorectal cancer treatment, and comorbidities. The relationship between all prescription nonanticancer drugs and the mortality of colorectal cancer patients was analyzed, controlling for multiple comparisons with the false discovery rate.

**Results:**

We found that one ATC level-2 drug (drugs that act on the nervous system, including parasympathomimetics, addictive disorder drugs, and antivertigo drugs) showed a protective effect related to colorectal cancer prognosis. At the ATC level 4 classification, 4 drugs were significant: two had a protective effect (anticholinesterases and opioid anesthetics), and the other two had a detrimental effect (magnesium compounds and Pregnen [4] derivatives).

**Conclusions:**

In this hypothesis-free study, we identified four drugs linked to colorectal cancer prognosis. The MWAS method can be useful in real-world data analysis.

**Supplementary Information:**

The online version contains supplementary material available at 10.1186/s12885-023-11105-9.

## Introduction

Colorectal cancer is the third most common cancer and the second leading cause of cancer death worldwide [1]. In addition, in high-HDI (human development index) countries, the survival rate of colorectal cancer has steadily increased due to early detection and improved treatment outcomes. As a result, the prevalence of colorectal cancer is steadily increasing [2]. Lifestyle factors such as drinking, smoking, obesity, and low physical activity are risk factors that act in common not only for colorectal cancer but also for noncancer chronic diseases. Chronic diseases such as hypertension, diabetes, and hyperlipidemia often accompany colorectal cancer [3, 4]. Because it is limited to detecting rare adverse effects through clinical trials, it is necessary to reveal unintended effects of drugs through pharmacovigilance after drug marketing [5]. Pharmacovigilance after drug marketing is mainly performed by monitoring reports of adverse events, but rare events with relatively long induction periods, such as cancer occurrence or cancer-caused death, are difficult to monitor [6]. In cases such as these, pharmacoepidemiological studies are helpful, as are representative examples of the relationship between noncancer chronic disease medications, including metformin (a treatment for type 2 diabetes), statins (a treatment for hyperlipidemia), and aspirin (which is known to be effective in preventing cardiovascular disease), and the survival prognosis of colorectal cancer patients [7–12]. However, these studies are hypothesis-driven, and if selective reporting occurs, biased results may be obtained [13, 14]. Recently, as real-world data such as health insurance claim data become available, hypothesis non-specific studies using the concept of hypothesis-free data-driven approach are being conducted. In the field of pharmacoepidemiology, which systematically analyzes the relationship between various drugs and outcomes, this type of study, modeled after the genome-wide association study (GWAS), is known as a medication-wide association study (MWAS) or a drug-wide association study (DWAS) [15–20]. Hypothesis-free studies prevent selective reporting of results by correcting for multiple comparisons in the analysis and reporting it transparently [13, 14, 20]. We analyzed the relationship between prescription drugs and mortality in patients diagnosed with colorectal cancer using the Korean National Health Insurance Service-National Sample Cohort database (KNHIS-NSC).

## Materials and methods

### Database

This study was conducted using data from individuals enrolled in the KNHIS-NSC from 2002 to 2015 [21]. The KNHIS-NSC database consists of claim data regarding the use of medical institutions such as hospitals and pharmacies by Korean National Health Insurance subscribers. All medical institutions in Korea are obliged to participate in the Korean National Health Insurance system. Therefore, claim data include data from all hospitals and pharmacies in Korea. These data, consisting of systematic stratified random sampling of 1,025,340 people (approximately 2% of the Korean population), were released for research purposes. The database includes demographic information such as sex, age, date of death, cause of death, income decile, and treatment date; diagnosis information based on the Korean Standard Classification of Diseases (KCD) reflecting the International Standard Classification of Diseases (ICD); records of inpatient and outpatient usage and related prescriptions; and health-examination results [21].

### Study sample

Colorectal cancer patients were defined as a case with at least one of the KCD codes C18, C19, and C20 who underwent diagnostic tests, such as colonoscopy, or received colorectal cancer treatment, such as surgery, chemotherapy, or radiation therapy [9, 22]. The date of the first treatment was set as time zero (i.e., study entry). To include only newly diagnosed colorectal cancer patients, a wash-out period of 2 years was set; therefore, patients diagnosed with colorectal cancer in 2002 and 2003 were excluded.

### Cross validation

**Fig. 1:**
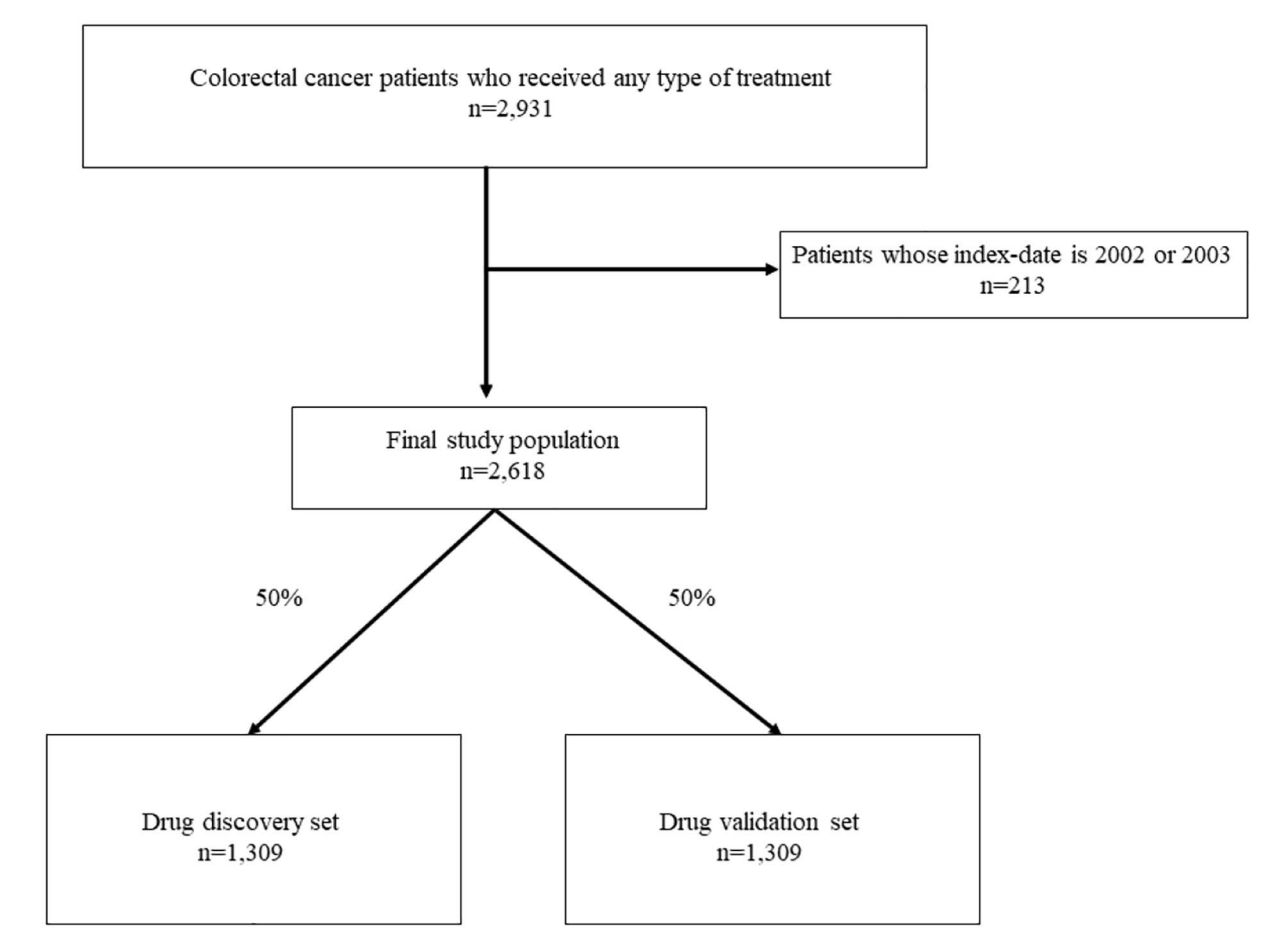
Study flow diagram

Cross validation was performed to ensure the reproducibility of the results. The selected participants were randomly divided into drug discovery and drug validation sets in a 1:1 ratio. Then, sequential analysis was performed using the two-stage method [23]. The selection process for the study population is depicted in Fig. 1.

### Exposure

**Fig. 2:**
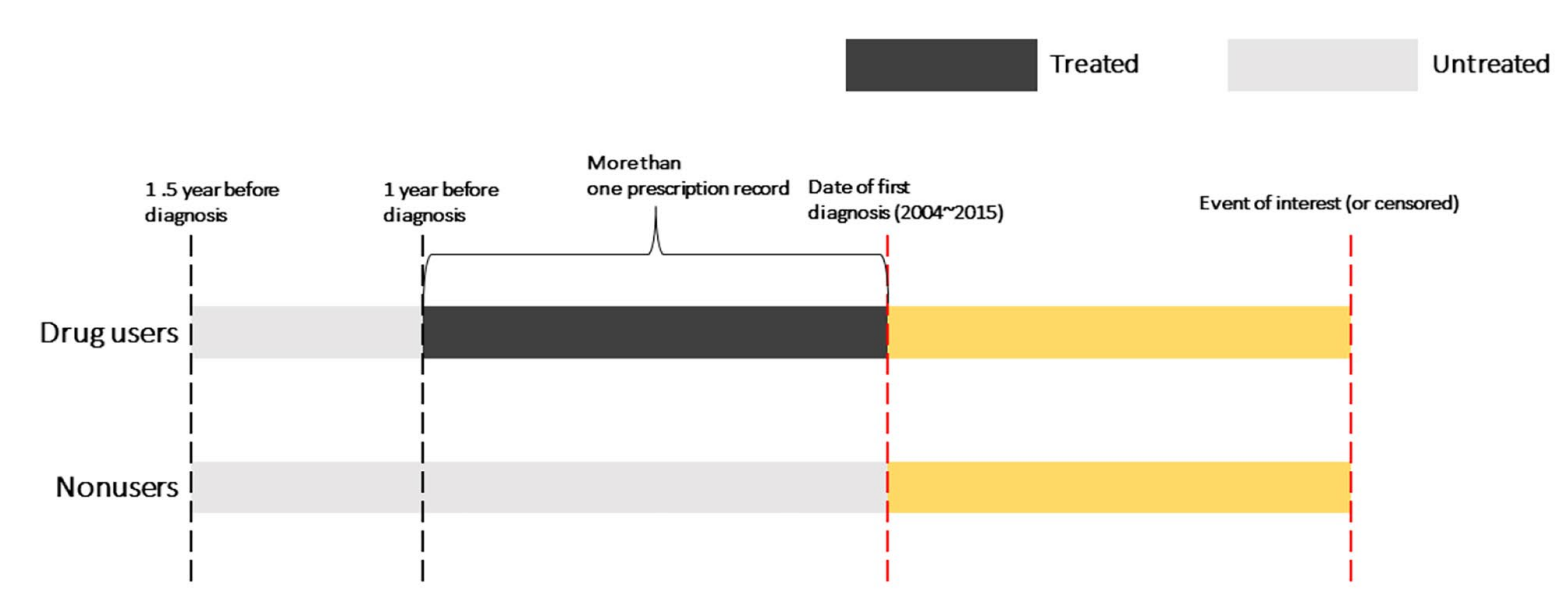
Schematic diagram of the definitions of drug users and nonusers

After drugs classified as anticancer drugs according to the Korean Ministry of Food and Drug Safety were excluded, all drug prescription data were converted to their Anatomical Therapeutic Chemical (ATC) Classification System codes. The ATC system is a drug classification system administered by the World Health Organization that classifies drugs into five levels. The ATC codes were analyzed at ATC levels 2 (therapeutic group) and 4 (chemical subgroup) [24]. In pharmacoepidemiological studies, if prevalent users are included, selection bias may occur. This is because prevalent users survive while taking drugs and may have improved health-related behaviors [25–28]. To prevent such selection bias, nonusers can be compared with incident users who had no history of the target drug use before study entry and started taking the target drugs after study entry [27, 28]. However, this method can also lead to confounding by indication (i.e., treatment is generally given to a patient with a specific condition) [27]. To solve this problem, we defined drug users as patients who did not take certain drugs for 1–1.5 years before study entry and then took drugs for the past 1 year before study entry (i.e., past 1-year incident users) (Fig. 2).

### Outcomes

We used the outcomes of all-cause mortality and colorectal cancer-specific mortality. Follow-up started at the date of the first treatment for colorectal cancer and terminated at whichever came first, the date of death or the date of the end of follow-up of the KNHIS-NSC (December 31, 2015).

### Covariates

We adjusted for the following covariates: sex, age, and Charlson Comorbidity Index (CCI). The treatment received by the patient, such as colonoscopy, surgery, chemotherapy, and radiation therapy, was included as a proxy variable for colorectal cancer staging as used in previous studies. We categorized the study population based on cancer treatment, considering that patients with early-stage cancer typically undergo surgery alone, while those with advanced-stage cancer often receive palliative therapy such as chemotherapy or radiotherapy without undergoing surgery [9, 22]. To adjust for patients who did not receive any treatment after colorectal cancer diagnosis (i.e., terminal stage), undergoing colonoscopy only was included as a covariate for treatment.

### Statistical analysis

**Fig. 3:**
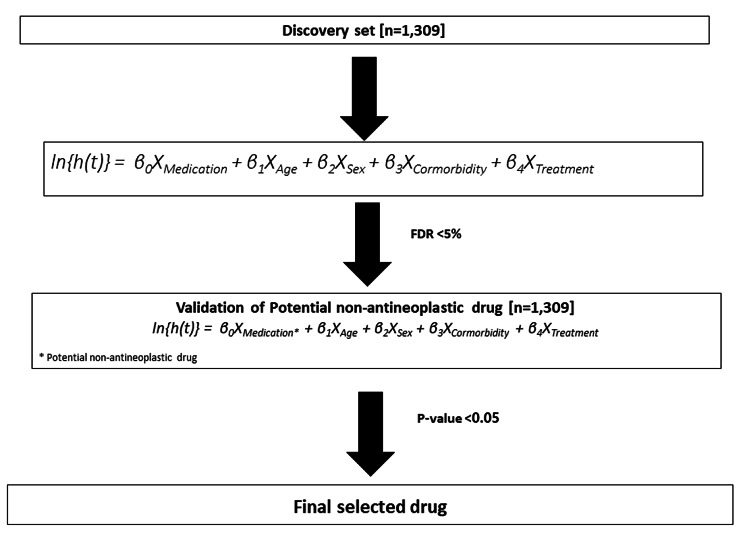
Summary of the analytic method

We performed a survival analysis using the Cox proportional hazards model. Because hypothesis-free studies analyze the relationship between many drugs and outcomes at once, type I errors (false positives) increase due to multiple comparisons. In this study, we controlled for type I errors using the false discovery rate (FDR). The FDR represents the proportion of falsely rejected hypotheses in multiple comparison [29]. Significant signals were first detected in the drug discovery set with an FDR of 5%, and then the result was subsequently verified with a p value threshold of 0.05 in the drug validation set (Fig. 3). The results of each drug’s verification were displayed using a volcano plot, which is a type of scatter plot. The x-axis was represented by the base-2 logarithm of the HR, while the y-axis was represented by the negative logarithm of the FDR-adjusted p value.

## Results

### Study characteristics

After excluding 213 patients who received colorectal cancer treatment in 2002 and 2003, which was set as the wash-out period, a total of 2,618 colorectal cancer patients met the eligibility criteria and were included in the study (Fig. 1). A total of 1,309 patients were included in the drug discovery set and the drug validation set. The characteristics of the study subjects are presented in Table 1. Overall, the distributions of the general characteristics of the two datasets were similar except for the higher proportion of males in the discovery set.

**Table 1:** General characteristics of the colorectal cancer patients included in the discovery and validation sets

Variable	Discovery set N = 1309	Validation set N = 1309
Sex, n (%)		
Male	864 (66.0)	805 (61.5)
Female	445 (34.0)	504 (38.5)
Age at diagnosis of colorectal cancer, n (%)		
15–39 years	48 (3.7)	38 (2.9)
40–49 years	138 (10.5)	141 (10.8)
50–59 years	299 (22.8)	297 (22.7))
60–69 years	392 (29.9)	384 (29.3)
70–79 years	345 (26.4)	331 (25.3)
> 79 years	87 (6.7)	118 (9.0)
Follow-up period, months		
Mean ± SD	53.8 ± 40.9	53.0 ± 40.5
Cancer treatment, n (%)		
Colonoscopy only	147 (11.2)	144 (11.0)
Operation only	254 (19.4)	244 (18.6)
Operation and chemotherapy	21 (1.6)	20 (1.5)
Operation and radiotherapy	343 (26.2)	358 (27.4)
Operation with both radiotherapy and chemotherapy	259 (19.8)	273 (20.9)
Radiotherapy or chemotherapy without operation	285 (21.8)	270 (20.6)
Charlson Comorbidity Index, n (%)		
0	100 (7.6)	94 (7.2)
1–2	408 (31.2)	464 (35.3)
3+	801 (61.2)	752 (57.5)

### Drugs classified as ATC level 2

**Fig. 4:**
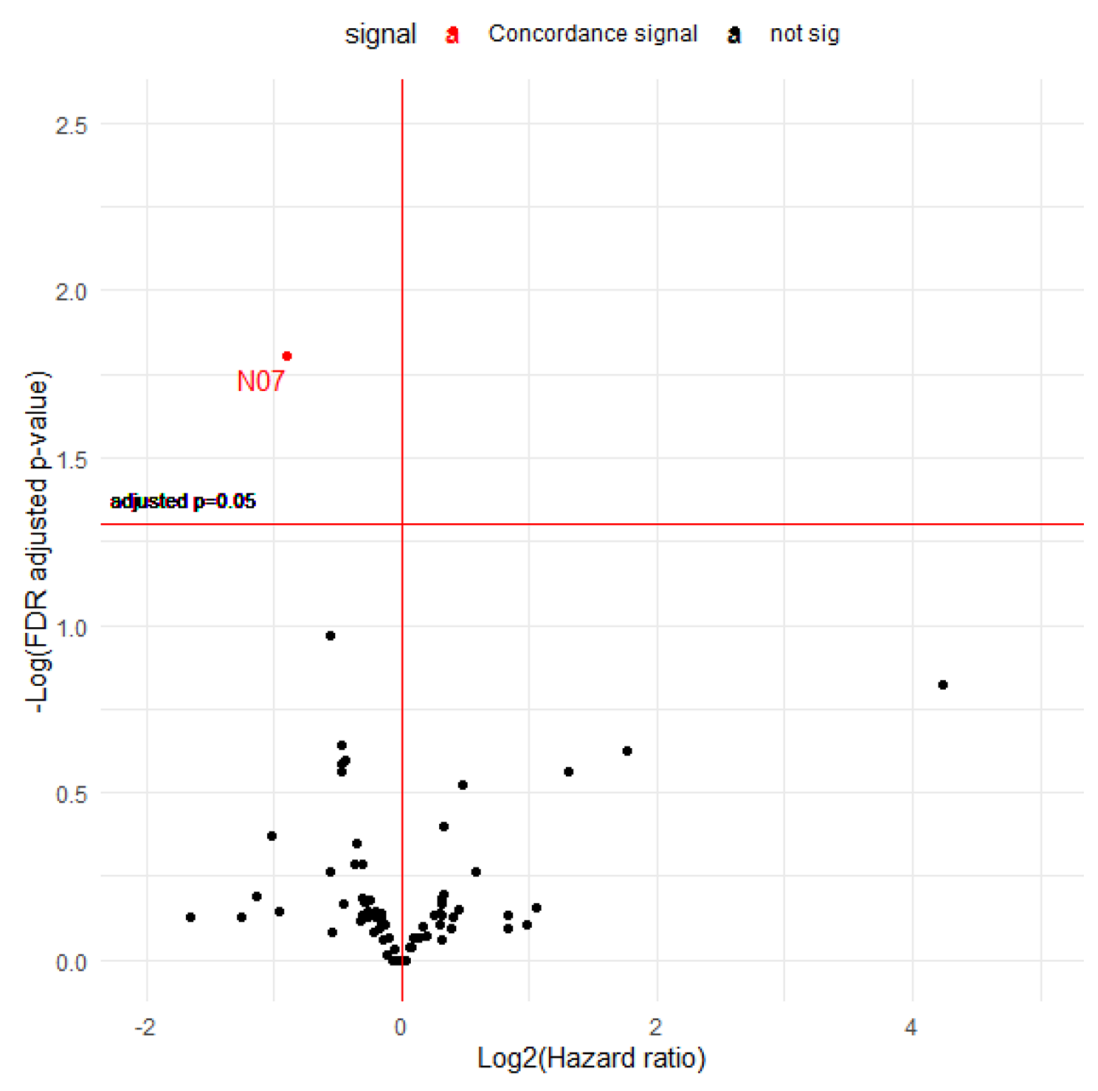
Volcano plot demonstrating the hazard ratios (HRs) and FDR-adjusted p values for the association between drugs classified as ATC level 2 and all-cause mortality

**Fig. 5:**
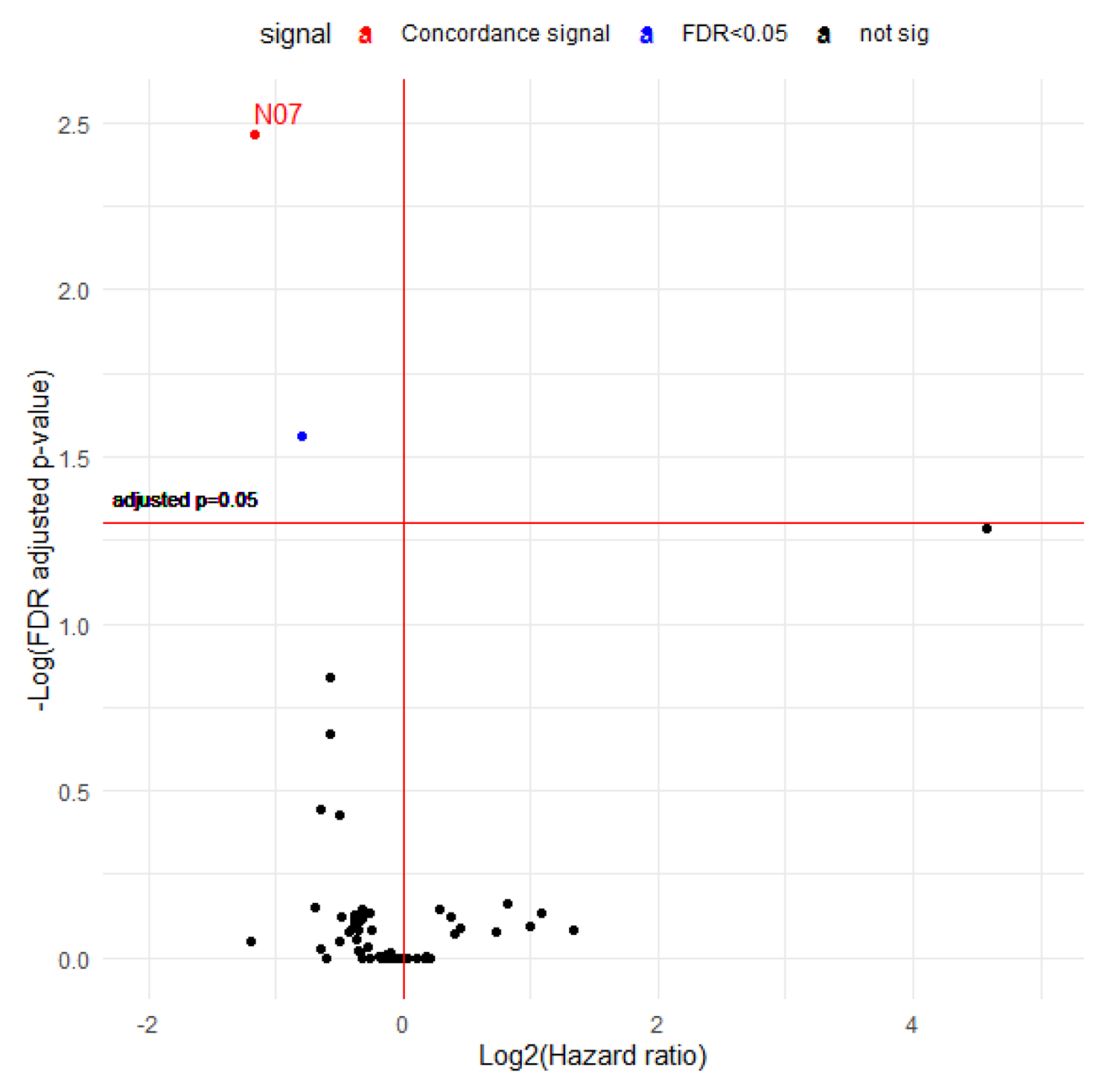
Volcano plot demonstrating the hazard ratios (HRs) and FDR-adjusted p values for the association between drugs classified as ATC level 2 and colorectal cancer-specific mortality

A total of 76 drugs classified as ATC level 2 were included in the analysis. A volcano plot of detected signals for all-cause mortality is presented in Fig. 4. Only one signal (N07: Other nervous system drugs) was found in the drug discovery set based on the preset 5% FDR. This drug had a protective effect that was reproduced in the drug validation set. Figure 5 displays the signal search results for colorectal cancer-specific mortality. Other nervous system drugs (N07) repeatedly exhibited significant signals. One signal (J01: Antibacterials for systemic use, protective effect) was additionally discovered in the drug discovery set but not reproduced in the validation set. The full results are presented in Supplementary Table 1.

### Drugs classified as ATC level 4

**Fig. 6:**
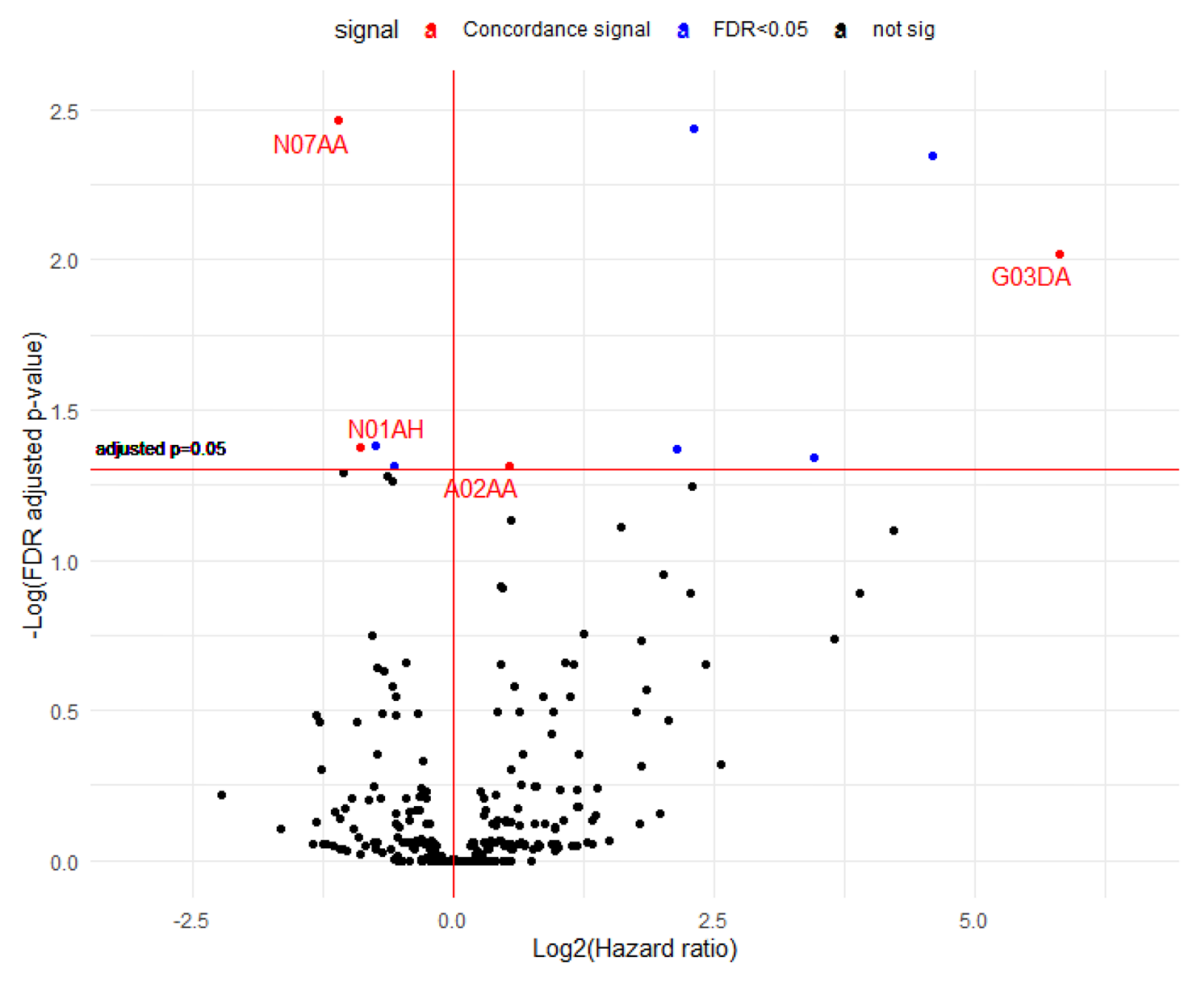
Volcano plot demonstrating the hazard ratios (HRs) and FDR-adjusted p values for the association between drugs classified as ATC level 4 and all-cause mortality

**Fig. 7:**
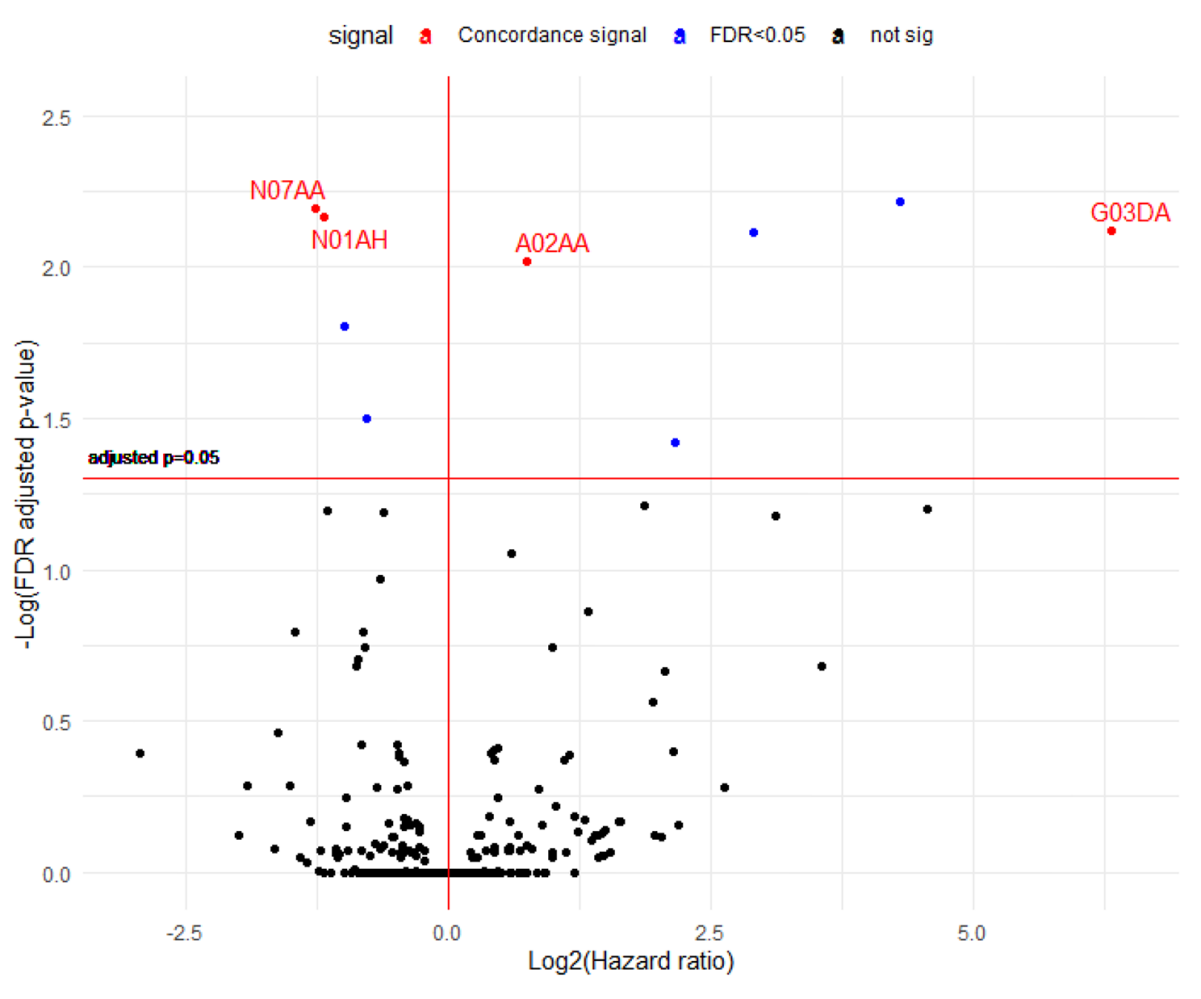
Volcano plot demonstrating the hazard ratios (HRs) and FDR-adjusted p values for the association between drugs classified as ATC level 4 and colorectal cancer-specific mortality

A total of 332 drugs classified as ATC level 4 were included in the analysis. As shown in Fig. 6, for all-cause mortality, ten drugs exceeded the threshold in the drug discovery set. Four of these drugs also produced significant results in the drug validation set. Two drugs exerted a protective effect (N07AA: anticholinesterases, N01AH: opioid anesthetics), and two drugs exerted a detrimental effect (magnesium compounds (A02AA) and Pregnen [4] derivatives (G03DA)). For colorectal cancer-specific mortality, a total of 9 drugs were significant in the drug discovery set, and 4 of these drugs were also significant in the validation set. The drugs that were significant in all-cause mortality were equally significant in colorectal cancer-specific mortality (Fig. 7). The full results are presented in Supplementary Table 2.

### Results for drugs previously reported to be associated with colorectal cancer prognosis

Next, we assessed whether metformin, statins, and aspirin, which have been reported to be related to the survival of colorectal cancer, showed a significant relationship with mortality in our study [7–12]. None of these drugs showed significant results even without FDR correction. Moreover, for the hazard ratio, the direction of the effect of metformin and statins was to increase mortality (metformin (A10BA): HR for all-cause mortality, 1.26; 95% CI, 0.71–2.24; HR for colorectal cancer-specific mortality, 1.48; 95% CI, 0.79–2.79; statin (C10AA): HR for all-cause mortality, 1.3; 95% CI, 0.85–1.98; HR for colorectal cancer-specific mortality, 1.05; 95% CI, 0.6–1.83). However, aspirin (B01AC) tended to decrease mortality (HR for all-cause mortality, 0.9; 95% CI, 0.57–1.40; HR for colorectal cancer-specific mortality, 0.84; 95% CI, 0.49–1.45) (Supplementary Table 2).

## Discussion

This study aimed to discover a new hypothesis related to the survival of colorectal cancer patients using a hypothesis-free method. In the analysis, a total of 4 drugs were identified to be related to the prognosis of colorectal cancer patients.

Anticholinesterases (N07AA) and opioid anesthetics (N01AH) exerted protective effects against both all-cause mortality and colorectal cancer-specific mortality. To the best of our knowledge, no epidemiological studies have reported a relationship between these two drugs and cancer. Although no in vitro or in vivo studies have found that anticholinesterases are directly related to colorectal cancer, several studies have shown that acetylcholine acts on colorectal cancer muscarinic receptor (MR) signaling to promote the proliferation of colorectal cancer cells [30–32]. Opioid anesthetics may be related to surgery under general anesthesia. Therefore, rather than opioid anesthetics themselves affecting the prognosis of colorectal cancer patients, it is possible that health-related behaviors after general anesthesia for surgery acted as a confounder. Two other drugs (Pregnen [4] derivatives and magnesium compounds) had a detrimental effect on the prognosis of colorectal cancer patients. Epidemiological studies have shown that progesterone from Pregnen [4] derivatives increases mortality in colorectal cancer patients. In the Women’s Health Initiative (WHI) trial, the incidence of colorectal cancer was lower in the group that took estrogen plus progestin than in the group that took the placebo, but the stage of cancer was more advanced at the time of diagnosis [33]. A follow-up study also showed that the hormone group exhibited more advanced stages of colorectal cancer and, although it was not significantly different, that the colorectal cancer mortality rate was also higher [34]. Magnesium compounds (A02AA) were a significant signal in colorectal cancer-specific mortality. No studies have found that magnesium compounds are related to the prognosis of colorectal cancer patients. Indeed, many epidemiological studies have reported that magnesium compounds lower the incidence of colorectal cancer [35–37]. Magnesium compounds may have different effects before and after the onset of colorectal cancer. Alternatively, although magnesium protects against colorectal cancer, magnesium intake in patients diagnosed with colorectal cancer may also have residual confounders that offset its protective effect. To interpret this signal, further studies on the effects of magnesium in patients with colorectal cancer are needed.

While metformin, statins, and aspirin were previously reported to be associated with the prognosis of colorectal cancer patients [7–12], this result was not reproduced in our study. These three drugs did not significantly influence colorectal cancer patient mortality even when the p values were not FDR corrected. Moreover, only aspirin had an effect in the predicted direction (to decrease mortality). These differences are thought to be due to differences in study design. The hypothesis-free method has the advantage of including all covariates in the model simultaneously and deriving results for various exposures. However, it also has several disadvantages, such as the lack of hypothesis-specific model selection and subgroup analysis. For example, studies that analyzed the prognosis of patients with metformin and colorectal cancer were conducted by limiting the subjects to diabetic patients or including patients’ adherence to metformin in the model [9, 11]. Additionally, the results can be influenced by the drug user definition (i.e., prevalent vs. incident users). In pharmacoepidemiological studies, it is recommended that only subjects whose exposure started at the time of follow-up be included in the study to prevent prevalent user bias [27, 28]. To avoid prevalent user bias and confounding by indication, the subjects were limited to past 1-year incident users in our study. In a study examining the relationship between statin use and cancer mortality, statin exposure and cancer-specific mortality were inversely associated in prevalent users, but this association disappeared in incident users; moreover, the direction of the effect was changed such that it increased cancer-specific mortality [38]. In addition, since the claim data used in this study reflects the real world, it should be considered that the subjects might have used the medications without control in various clinical environments.

The strength of this study is that it attempted to identify drugs related to the prognosis of cancer patients with a hypothesis-free method. Previous MWASs or DWASs have focused on drugs related to the incidence of cancer [18–20]. In particular, it was proposed to define drug users as past year incident users, thereby minimizing prevalent user bias and confounding by indications. In addition, the past year serves as a landmark time that provides effective control over the immortal-time bias. Additionally, a new hypothesis was presented as in the previously hypothesis-free studies; specifically, in this study, we found a total of 4 signals related to colorectal cancer prognosis. Presenting the signals in this manner can prevent selective reporting, as all results are systematically derived under the control of multiple comparisons. Further studies are needed to determine whether these associations are causal. The limitations of this study are as follows. First, since this study is an observational study, it cannot be free from the issue of validity due to comparability [39]. In the hypothesis-free approach, since many hypotheses are tested at once, if there is a problem with the validity of the study, it can lead to more serious issues. Unlike clinical trials that randomly assign individuals to take or not take the drug, basic differences between drug users and nonusers in hypothesis-free studies may generate comparability problems [40]. This problem is more serious when the indication of the drug is related to the patient’s prognosis. To solve this problem, we restricted the definition of drug use to 1 year before receiving colorectal cancer treatment and adjusted for the type of treatment received by colorectal cancer patients as well as the CCI. In a study that used the same data as this study, the type of treatment received by colorectal cancer patients was reported to be a useful proxy variable for cancer staging [9, 22]. Second, although the KNHIS-NSC data are representative of the Korean population, they were not collected for research purposes but are real-world data from health insurance claims. Therefore, information bias may occur. However, in the case of cancer, the accuracy of the diagnostic code is relatively high, and the risk of misclassification was reduced through the operational definition, including the type of colorectal cancer treatment [41].

## Conclusion

In this study, we found four signals related to prognosis in colorectal cancer patients through a hypothesis-free method. In addition, we observed conflicting results for statin, metformin, and aspirin compared to those of previous studies. The MWAS approach can be useful for pharmacovigilance in rare events with a long induction period and for proposing a new hypothesis.

## Electronic supplementary material

Below is the link to the electronic supplementary material.

Supplementary Material 1

## Data Availability

The NHIS-NSC data used to support the findings of this study are only available to authorized personnel and can be accessed from the website of the NHIS (https://nhiss.nhis.or.kr) after completing the application process and receiving approval (http://nhiss.nhis.or.kr/bd/ab/bdaba021eng.do).

## Abbreviations

ATC: Anatomical therapeutic chemical classification system
CCI: Charlson comorbidity index
DWAS: Drug-wide association study
FDR: False discovery rate
GWAS: Genome-wide association study
HDI: Human development index
HR: Hazard ratio
ICD: International standard classification of diseases
KCD: Korean standard classification of diseases
KNHIS-NSC: Korean national health insurance service-national sample cohort
MR: Muscarinic receptor
MWAS: Medication-wide association study
WHI: Women’s health initiative
