# Supplementary material for: The association between prescription drugs and colorectal cancer prognosis: a nationwide cohort study using a medication-wide association study
Source: BMC Cancer. 2023 Jul 10;23:643. doi: 10.1186/s12885-023-11105-9 (PMC10334631; doi:10.1186/s12885-023-11105-9)
Supplement: Supplementary file 1 — Supplementary Material 1 [file 12885_2023_11105_MOESM1_ESM.docx]

Supplementary Table 1. Hazard ratios and p values for all ATC level 2 drugs included in the analysis

| ATC | All-cause mortality | | | | | Colorectal cancer-specific mortality | | | | |
| --- | --- | --- | --- | --- | --- | --- | --- | --- | --- | --- |
|  | HR (95% CI) | p value | | FDR | Replication in  the drug validation set | HR (95% CI) | p value | | FDR | Replication in the  drug validation set |
| A01 | 0.99 (0.59-1.66) | 0.96171 | | 0.04671 | No | 0.79 (0.42-1.5) | 0.47730 | | 0.02566 | No |
| A02 | 1.09 (0.76-1.58) | | 0.63586 | 0.0375 | No | 1.03 (0.66-1.59) | 0.90222 | | 0.03947 | No |
| A03 | 0.73 (0.45-1.17) | | 0.1931 | 0.01447 | No | 0.62 (0.36-1.06) | 0.08093 | | 0.00592 | No |
| A04 | 1.12 (0.79-1.58) | | 0.52404 | 0.03355 | No | 0.95 (0.63-1.45) | 0.82738 | | 0.03684 | No |
| A05 | 1.39 (1.02-1.91) | | 0.03884 | 0.00658 | No | 1.3 (0.89-1.88) | 0.17276 | | 0.01184 | No |
| A06 | 0.74 (0.57-0.95) | | 0.0197 | 0.00395 | No | 0.93 (0.68-1.28) | 0.66682 | | 0.03421 | No |
| A07 | 0.99 (0.79-1.25) | | 0.95486 | 0.04605 | No | 0.93 (0.71-1.22) | 0.60056 | | 0.03224 | No |
| A09 | 1 (0.55-1.82) | | 0.99406 | 0.05 | No | 0.95 (0.47-1.93) | 0.88996 | | 0.03882 | No |
| A10 | 1.49 (0.91-2.44) | | 0.11254 | 0.01053 | No | 1.37 (0.76-2.45) | 0.29706 | | 0.01908 | No |
| A11 | 0.93 (0.67-1.3) | | 0.67857 | 0.04013 | No | 0.64 (0.42-0.97) | 0.03638 | | 0.00526 | No |
| A12 | 0.98 (0.65-1.46) | | 0.90027 | 0.04474 | No | 0.92 (0.58-1.46) | 0.71838 | | 0.03487 | No |
| A16 | 0.8 (0.44-1.46) | | 0.46607 | 0.03092 | No | 0.8 (0.39-1.62) | 0.53625 | | 0.02763 | No |
| B01 | 0.87 (0.62-1.22) | | 0.42255 | 0.02961 | No | 0.77 (0.51-1.15) | 0.19590 | | 0.01316 | No |
| B02 | 0.89 (0.68-1.17) | | 0.40043 | 0.02697 | No | 0.79 (0.57-1.08) | 0.13559 | | 0.00855 | No |
| B03 | 1.19 (0.79-1.81) | | 0.41021 | 0.02829 | No | 0.75 (0.42-1.34) | 0.32632 | | 0.02039 | No |
| B05 | 1.02 (0.79-1.31) | | 0.88487 | 0.04408 | No | 1.02 (0.76-1.36) | 0.92146 | | 0.04013 | No |
| C01 | 0.78 (0.58-1.05) | | 0.10122 | 0.00987 | No | 0.8 (0.56-1.13) | 0.19964 | | 0.01382 | No |
| C02 | 1.32 (0.78-2.22) | | 0.29859 | 0.02039 | No | 1.32 (0.72-2.42) | 0.37411 | | 0.02303 | No |
| C03 | 1.24 (0.92-1.68) | | 0.16102 | 0.0125 | No | 1.13 (0.79-1.61) | 0.50114 | | 0.02632 | No |
| C04 | 0.81 (0.55-1.18) | | 0.26763 | 0.01842 | No | 0.7 (0.44-1.12) | 0.13551 | | 0.00789 | No |
| C05 | 0.88 (0.65-1.19) | | 0.41378 | 0.02895 | No | 1 (0.71-1.41) | 0.99905 | | 0.05 | No |
| C07 | 0.83 (0.54-1.28) | | 0.40347 | 0.02763 | No | 0.82 (0.5-1.36) | 0.44435 | | 0.025 | No |
| C08 | 0.83 (0.59-1.17) | | 0.2988 | 0.02105 | No | 0.88 (0.6-1.3) | 0.52456 | | 0.02697 | No |
| C09 | 1.23 (0.82-1.85) | | 0.32113 | 0.02237 | No | 1.15 (0.7-1.91) | 0.57842 | | 0.02961 | No |
| C10 | 1.26 (0.84-1.88) | | 0.26861 | 0.01908 | No | 0.98 (0.57-1.68) | 0.93689 | | 0.04079 | No |
| D01 | 0.68 (0.43-1.08) | | 0.09917 | 0.00921 | No | 0.88 (0.54-1.42) | 0.58819 | | 0.03092 | No |
| D02 | 0.32 (0.04-2.25) | | 0.25062 | 0.01711 | No | 0.44 (0.06-3.11) | 0.40715 | | 0.02368 | No |
| D03 | 1.97 (0.27-14.16) | | 0.50037 | 0.03224 | No | - | 0.97663 | | 0.04605 | No |
| D06 | 0.91 (0.59-1.39) | | 0.65137 | 0.03816 | No | 0.72 (0.41-1.25) | 0.23899 | | 0.01645 | No |
| D07 | 0.81 (0.59-1.12) | | 0.20975 | 0.01579 | No | 0.77 (0.52-1.13) | 0.17863 | | 0.0125 | No |
| D08 | 1(0. 41-2.43) | | 0.99398 | 0.04934 | No | 0.92 (0.34-2.5) | 0.87553 | | 0.0375 | No |
| D11 | 0.95 (0.24-3.85) | | 0.94677 | 0.04539 | No | - | 0.97182 | | 0.04342 | No |
| G01 | 1.37 (0.71-2.63) | | 0.34625 | 0.025 | No | 1.02 (0.47-2.22) | 0.96158 | | 0.04145 | No |
| G02 | - | | 0.9697 | 0.04803 | No | - | 0.97294 | | 0.04474 | No |
| G03 | 0.92 (0.45-1.89) | | 0.82126 | 0.04342 | No | 0.83 (0.36-1.9) | 0.66095 | | 0.03355 | No |
| G04 | 1.24 (0.88-1.74) | | 0.22107 | 0.01645 | No | 1.12 (0.74-1.7) | 0.58027 | | 0.03026 | No |
| H01 | 1.78 (0.66-4.79) | | 0.25596 | 0.01776 | No | 1.03 (0.25-4.15) | 0.97199 | | 0.04408 | No |
| H02 | 0.81 (0.62-1.05) | | 0.1138 | 0.01118 | No | 0.71 (0.52-0.97) | 0.03303 | | 0.00461 | No |
| H03 | 0.51 (0.13-2.06) | | 0.34563 | 0.02434 | No | 0.66 (0.16-2.67) | 0.56118 | | 0.02895 | No |
| H04 | 18.79 (2.56-137.75) | | 0.00391 | 0.00132 | No | 23.76 (3.19-176.91) | | 0.00198 | 0.00197 | No |
| H05 | 0.42 (0.06-3.01) | | 0.38844 | 0.02632 | No | - | 0.96622 | | 0.04276 | No |
| J01 | 0.72 (0.55-0.95) | | 0.02068 | 0.00461 | No | 0.58 (0.42-0.79) | 0.00070 | | 0.00132 | No |
| J02 | 0.86 (0.52-1.42) | | 0.55444 | 0.03421 | No | 1.01 (0.58-1.76) | 0.98324 | | 0.04803 | No |
| J04 | 2.09 (0.67-6.55) | | 0.2075 | 0.01513 | No | 1.99 (0.49-8.06) | 0.33448 | | 0.02171 | No |
| J05 | 0.5 (0.23-1.05) | | 0.06626 | 0.00789 | No | 0.1 (0.01-0.7) | 0.02051 | | 0.00395 | No |
| J06 | 0.69 (0.17-2.76) | | 0.59681 | 0.03684 | No | - | 0.96539 | | 0.04211 | No |
| M01 | 0.72 (0.53-0.97) | | 0.03048 | 0.00592 | No | 0.8 (0.56-1.14) | 0.20825 | | 0.01513 | No |
| M02 | 0.88 (0.58-1.36) | | 0.57117 | 0.03618 | No | 0.77 (0.45-1.3) | 0.32726 | | 0.02105 | No |
| M03 | 0.83 (0.64-1.08) | | 0.15652 | 0.01184 | No | 0.79 (0.58-1.08) | 0.13722 | | 0.00921 | No |
| M04 | 2.48 (1.1-5.57) | | 0.02828 | 0.00526 | No | 2.12 (0.79-5.71) | 0.13856 | | 0.00987 | No |
| M05 | 1.14 (0.6-2.18) | | 0.68373 | 0.04079 | No | 1 (0.44-2.29) | 0.99362 | | 0.04934 | No |
| M09 | 0.68 (0.52-0.89) | | 0.00419 | 0.00197 | No | 0.67 (0.49-0.92) | 0.01352 | | 0.00329 | No |
| N01 | 0.84 (0.66-1.08) | | 0.17188 | 0.01316 | No | 0.67 (0.5-0.9) | 0.00733 | | 0.00263 | No |
| N02 | 0.87 (0.66-1.15) | | 0.32463 | 0.02303 | No | 0.84 (0.61-1.16) | 0.29171 | | 0.01842 | No |
| N03 | 1.24 (0.82-1.87) | | 0.3138 | 0.02171 | No | 1.08 (0.64-1.83) | 0.77712 | | 0.03553 | No |
| N04 | 1.79 (0.25-12.76) | | 0.56367 | 0.03553 | No | 2.53 (0.35-18.11) | 0.35667 | | 0.02237 | No |
| N05 | 0.72 (0.56-0.94) | | 0.01426 | 0.00263 | No | 0.83 (0.61-1.13) | 0.24173 | | 0.01711 | No |
| N06 | 1.05 (0.76-1.47) | | 0.75808 | 0.04211 | No | 1.12 (0.77-1.65) | 0.55254 | | 0.02829 | No |
| N07 | 0.54 (0.39-0.75) | | 0.0002 | 0.00066 | Yes | 0.45 (0.3-0.66) | 0.00004 | | 0.00066 | Yes |
| P01 | 0.46 (0.15-1.42) | | 0.17549 | 0.01382 | No | 0.64 (0.2-1.99) | 0.43729 | | 0.02434 | No |
| P02 | - | | 0.97987 | 0.04868 | No | - | 0.98371 | | 0.04868 | No |
| R01 | 1.06 (0.8-1.41) | | 0.66812 | 0.03947 | No | 1.03 (0.73-1.43) | 0.87978 | | 0.03816 | No |
| R02 | 0.96 (0.74-1.24) | | 0.75428 | 0.04145 | No | 0.8 (0.58-1.09) | 0.15465 | | 0.01053 | No |
| R03 | 0.9 (0.68-1.18) | | 0.43026 | 0.03026 | No | 0.81 (0.59-1.12) | 0.20777 | | 0.01447 | No |
| R05 | 0.87 (0.66-1.13) | | 0.2884 | 0.01974 | No | 0.78 (0.57-1.07) | 0.12096 | | 0.00658 | No |
| R06 | 0.78 (0.6-1.03) | | 0.07548 | 0.00855 | No | 0.78 (0.57-1.07) | 0.12209 | | 0.00724 | No |
| R07 | 1.23 (0.69-2.2) | | 0.48831 | 0.03158 | No | 1.01 (0.47-2.16) | 0.97784 | | 0.04737 | No |
| S01 | 0.91 (0.69-1.2) | | 0.50768 | 0.03289 | No | 0.92 (0.66-1.26) | 0.59090 | | 0.03158 | No |
| S02 | 1.31 (0.54-3.16) | | 0.55557 | 0.03487 | No | 1.76 (0.73-4.28) | 0.21005 | | 0.01579 | No |
| S03 | - | | 0.96949 | 0.04737 | No | - | 0.97461 | | 0.04539 | No |
| V03 | 0.81 (0.53-1.25) | | 0.34476 | 0.02368 | No | 0.76 (0.47-1.25) | 0.27728 | | 0.01776 | No |
| V04 | 1.24 (0.46-3.34) | | 0.66616 | 0.03882 | No | 1.65 (0.62-4.45) | 0.31901 | | 0.01974 | No |
| V06 | 3.41 (1.27-9.21) | | 0.01532 | 0.00329 | No | 0.97 (0.14-6.95) | 0.97703 | | 0.04671 | No |
| V07 | 1.05 (0.76-1.45) | | 0.76805 | 0.04276 | No | 0.92 (0.63-1.34) | 0.65475 | | 0.03289 | No |
| V08 | 0.89 (0.69-1.16) | | 0.38062 | 0.02566 | No | 0.96 (0.7-1.32) | 0.82155 | | 0.03618 | No |
| V09 | 1.25 (0.99-1.58) | | 0.057 | 0.00724 | No | 1.22 (0.93-1.59) | 0.15474 | | 0.01118 | No |

Supplementary Table 2. Hazard ratios and p values for all ATC level 4 drugs included in the analysis

| ATC | All-cause mortality | | | | Colorectal cancer-specific mortality | | | |
| --- | --- | --- | --- | --- | --- | --- | --- | --- |
|  | HR (95% CI) | p value | FDR | Replication in the  drug validation set | HR (95% CI) | p value | FDR | Replication in the  drug validation set |
| A01AC | 0.68 (0.17-2.73) | 0.581671 | 0.029669 | No | 0.93 (0.23-3.78) | 0.922782 | 0.039307 | No |
| A01AD | 0.98 (0.57-1.68) | 0.934666 | 0.041867 | No | 0.76 (0.39-1.48) | 0.41098 | 0.020482 | No |
| A02AA | 1.45 (1.15-1.82) | 0.001472 | 0.001506 | Yes | 1.68 (1.29-2.18) | 0.00011 | 0.000602 | Yes |
| A02AB | 1.37 (0.75-2.51) | 0.303232 | 0.01747 | No | 1.59 (0.82-3.1) | 0.173165 | 0.011295 | No |
| A02AC | 0.92 (0.43-1.94) | 0.821974 | 0.037952 | No | 0.74 (0.28-2) | 0.558386 | 0.026958 | No |
| A02AD | 0.92 (0.72-1.19) | 0.529586 | 0.027711 | No | 0.99 (0.74-1.33) | 0.96408 | 0.040361 | No |
| A02BA | 0.94 (0.72-1.21) | 0.624553 | 0.031325 | No | 0.89 (0.66-1.21) | 0.454225 | 0.021837 | No |
| A02BB | 0.81 (0.38-1.72) | 0.580168 | 0.029518 | No | 0.72 (0.3-1.77) | 0.476198 | 0.022741 | No |
| A02BC | 1.23 (0.94-1.62) | 0.13437 | 0.010693 | No | 1.36 (1-1.86) | 0.050417 | 0.005873 | No |
| A02BX | 1.38 (1.09-1.74) | 0.007081 | 0.002861 | No | 1.33 (1.01-1.74) | 0.043989 | 0.005422 | No |
| A03AA | 1.01 (0.8-1.29) | 0.924074 | 0.041416 | No | 1.21 (0.93-1.59) | 0.160956 | 0.010392 | No |
| A03AB | 0.64 (0.49-0.85) | 0.001662 | 0.001657 | No | 0.64 (0.47-0.87) | 0.004951 | 0.002259 | No |
| A03AC | 1.03 (0.82-1.3) | 0.7887 | 0.036145 | No | 1.11 (0.85-1.47) | 0.441737 | 0.021536 | No |
| A03AD | - | 0.964854 | 0.04503 | No | - | 0.969635 | 0.04247 | No |
| A03AE | 2.21 (0.31-15.86) | 0.431521 | 0.024247 | No | 2.79 (0.39-20.17) | 0.308431 | 0.017319 | No |
| A03AX | 0.96 (0.77-1.2) | 0.709234 | 0.033735 | No | 0.99 (0.76-1.29) | 0.932711 | 0.039759 | No |
| A03BA | 0.6 (0.35-1.04) | 0.06791 | 0.00753 | No | 0.36 (0.17-0.78) | 0.0088 | 0.002711 | No |
| A03BB | 0.84 (0.67-1.04) | 0.115237 | 0.009789 | No | 0.83 (0.64-1.07) | 0.148974 | 0.00994 | No |
| A03CA | 0.47 (0.07-3.39) | 0.456238 | 0.025151 | No | 0.65 (0.09-4.67) | 0.668546 | 0.030723 | No |
| A03FA | 1.02 (0.78-1.34) | 0.879788 | 0.040663 | No | 0.9 (0.66-1.23) | 0.522433 | 0.025301 | No |
| A04AA | 1.1 (0.7-1.75) | 0.673971 | 0.032831 | No | 0.81 (0.46-1.43) | 0.468639 | 0.02259 | No |
| A04AD | 1.23 (0.79-1.93) | 0.362397 | 0.020783 | No | 1.08 (0.61-1.91) | 0.785183 | 0.034789 | No |
| A05AA | 1.48 (0.95-2.3) | 0.084981 | 0.008434 | No | 1.27 (0.74-2.18) | 0.389892 | 0.019578 | No |
| A05AX | 1.06 (0.57-2) | 0.849274 | 0.039307 | No | 0.99 (0.47-2.09) | 0.972077 | 0.044277 | No |
| A05BA | 1.51 (1.05-2.17) | 0.025694 | 0.004819 | No | 1.28 (0.83-2) | 0.265872 | 0.015512 | No |
| A06AB | 0.92 (0.65-1.31) | 0.656188 | 0.03253 | No | 0.77 (0.5-1.19) | 0.239939 | 0.013855 | No |
| A06AC | 1.14 (0.87-1.48) | 0.346376 | 0.019729 | No | 1.23 (0.91-1.66) | 0.171531 | 0.011145 | No |
| A06AD | 0.68 (0.53-0.85) | 0.000982 | 0.001054 | No | 0.77 (0.58-1.02) | 0.066764 | 0.006476 | No |
| A06AG | 1.16 (0.72-1.87) | 0.539889 | 0.028614 | No | 1.35 (0.81-2.24) | 0.251801 | 0.014608 | No |
| A07AA | 1.58 (0.97-2.59) | 0.069066 | 0.007681 | No | 1.99 (1.17-3.39) | 0.010947 | 0.003012 | No |
| A07AX | 1.18 (0.38-3.7) | 0.775628 | 0.035843 | No | 1.5 (0.48-4.72) | 0.487556 | 0.023042 | No |
| A07BC | 1.22 (0.91-1.65) | 0.185603 | 0.013102 | No | 1.13 (0.79-1.61) | 0.520642 | 0.025151 | No |
| A07DA | 1.1 (0.78-1.55) | 0.601746 | 0.030422 | No | 1.22 (0.82-1.81) | 0.328596 | 0.018373 | No |
| A07EC | - | 0.971646 | 0.04759 | No | - | 0.973959 | 0.046235 | No |
| A07FA | 0.98 (0.78-1.22) | 0.843575 | 0.038855 | No | 0.93 (0.71-1.21) | 0.564385 | 0.02741 | No |
| A09AA | 1.21 (0.66-2.22) | 0.529943 | 0.027861 | No | 1.14 (0.56-2.31) | 0.723688 | 0.03238 | No |
| A10AB | 1.34 (0.86-2.09) | 0.203384 | 0.013705 | No | 1.22 (0.72-2.07) | 0.455727 | 0.021988 | No |
| A10AD | 3.37 (1.08-10.54) | 0.03681 | 0.005572 | No | 1.5 (0.21-10.72) | 0.686524 | 0.031325 | No |
| A10AE | 4.44 (1.83-10.79) | 0.000986 | 0.001205 | No | 3.88 (1.23-12.18) | 0.020333 | 0.003765 | No |
| A10BA | 1.26 (0.71-2.24) | 0.439847 | 0.024699 | No | 1.48 (0.79-2.79) | 0.22588 | 0.013253 | No |
| A10BB | 1.81 (1.06-3.1) | 0.030345 | 0.005271 | No | 1.82 (0.96-3.43) | 0.065455 | 0.006175 | No |
| A10BD | 1.19 (0.49-2.89) | 0.702433 | 0.033434 | No | 0.78 (0.19-3.13) | 0.720306 | 0.032078 | No |
| A10BF | 0.99 (0.44-2.23) | 0.979849 | 0.048946 | No | 0.79 (0.25-2.49) | 0.691513 | 0.031476 | No |
| A10BG | 0.42 (0.06-3) | 0.388257 | 0.022139 | No | 0.56 (0.08-4.01) | 0.564696 | 0.02756 | No |
| A10BH | 0.87 (0.22-3.52) | 0.849201 | 0.039157 | No | 1.22 (0.3-4.91) | 0.783042 | 0.034488 | No |
| A10BX | 4.82 (1.54-15.12) | 0.007023 | 0.002711 | No | 4.43 (1.1-17.95) | 0.036881 | 0.004669 | No |
| A11CC | - | 0.967018 | 0.045331 | No | - | 0.97173 | 0.043976 | No |
| A11DA | 1.47 (0.69-3.15) | 0.322136 | 0.018223 | No | 0.61 (0.15-2.48) | 0.491102 | 0.023343 | No |
| A11EA | 0.81 (0.54-1.2) | 0.285062 | 0.016717 | No | 0.63 (0.38-1.03) | 0.065858 | 0.006325 | No |
| A11GA | 0.86 (0.58-1.27) | 0.440526 | 0.024849 | No | 0.68 (0.42-1.1) | 0.114228 | 0.007982 | No |
| A11GB | - | 0.968569 | 0.046084 | No | - | 0.972581 | 0.044578 | No |
| A11HA | 1.03 (0.46-2.32) | 0.940451 | 0.042169 | No | 1.05 (0.43-2.55) | 0.922397 | 0.039157 | No |
| A12AA | 0.91 (0.48-1.71) | 0.765332 | 0.03509 | No | 1.04 (0.53-2.03) | 0.913888 | 0.038855 | No |
| A12AX | 0.87 (0.41-1.86) | 0.715105 | 0.033886 | No | 0.48 (0.15-1.5) | 0.206022 | 0.012199 | No |
| A12BA | 2.22 (1.13-4.32) | 0.019827 | 0.004367 | No | 2.03 (0.9-4.6) | 0.088527 | 0.007229 | No |
| A12CC | 0.83 (0.44-1.57) | 0.571869 | 0.029367 | No | 0.81 (0.4-1.65) | 0.559028 | 0.027108 | No |
| A16AA | 0.43 (0.06-3.06) | 0.398179 | 0.022741 | No | - | 0.972919 | 0.04503 | No |
| A16AX | 0.88 (0.47-1.66) | 0.692839 | 0.032982 | No | 0.97 (0.48-1.96) | 0.924692 | 0.039458 | No |
| B01AA | 0.53 (0.17-1.67) | 0.280228 | 0.016566 | No | 0.25 (0.04-1.79) | 0.166946 | 0.010843 | No |
| B01AB | 0.88 (0.59-1.32) | 0.533137 | 0.028163 | No | 0.81 (0.51-1.3) | 0.379815 | 0.019127 | No |
| B01AC | 0.9 (0.57-1.4) | 0.629825 | 0.031627 | No | 0.84 (0.49-1.45) | 0.522851 | 0.025753 | No |
| B01AX | 0.76 (0.36-1.62) | 0.481617 | 0.026807 | No | 0.4 (0.13-1.26) | 0.117762 | 0.008283 | No |
| B02AA | 0.96 (0.68-1.35) | 0.811856 | 0.037651 | No | 0.81 (0.53-1.22) | 0.310422 | 0.01747 | No |
| B02AB | 1.22 (0.63-2.37) | 0.562965 | 0.029217 | No | 1.15 (0.54-2.44) | 0.724352 | 0.032681 | No |
| B02BA | 0.59 (0.38-0.9) | 0.013396 | 0.003765 | No | 0.54 (0.33-0.89) | 0.014436 | 0.003464 | No |
| B02BC | 2.37 (1.21-4.65) | 0.012187 | 0.003464 | No | 2.24 (1.04-4.82) | 0.0398 | 0.00497 | No |
| B02BX | 0.63 (0.42-0.94) | 0.022121 | 0.004669 | No | 0.55 (0.34-0.88) | 0.012444 | 0.003163 | No |
| B03AA | 1 (0.56-1.78) | 0.998498 | 0.05 | No | 0.75 (0.35-1.59) | 0.453566 | 0.021687 | No |
| B03AB | 1.06 (0.39-2.86) | 0.909638 | 0.040964 | No | 0.61 (0.15-2.45) | 0.482634 | 0.022892 | No |
| B03AC | 1.27 (0.18-9.08) | 0.809964 | 0.0375 | No | - | 0.96564 | 0.040663 | No |
| B03AD | 1.49 (0.48-4.66) | 0.493043 | 0.027108 | No | 1.51 (0.37-6.1) | 0.563372 | 0.027259 | No |
| B03BA | 1.25 (0.4-3.92) | 0.697952 | 0.033133 | No | 0.58 (0.08-4.12) | 0.583433 | 0.027861 | No |
| B03BB | 2.31 (0.95-5.65) | 0.066463 | 0.00738 | No | 0.5 (0.07-3.6) | 0.493655 | 0.023645 | No |
| B03XA | 1.96 (0.63-6.12) | 0.247323 | 0.015663 | No | 0.79 (0.11-5.65) | 0.814552 | 0.035392 | No |
| B05AA | 0.99 (0.68-1.43) | 0.953205 | 0.042771 | No | 0.92 (0.6-1.39) | 0.677353 | 0.030873 | No |
| B05BA | 0.84 (0.67-1.06) | 0.135656 | 0.010843 | No | 0.65 (0.5-0.86) | 0.002232 | 0.001807 | No |
| B05BB | 0.95 (0.76-1.19) | 0.642684 | 0.032229 | No | 0.86 (0.66-1.12) | 0.257466 | 0.01506 | No |
| B05BC | 1.6 (0.51-5.02) | 0.416377 | 0.023645 | No | 0.67 (0.09-4.82) | 0.694753 | 0.031777 | No |
| B05CA | - | 0.968044 | 0.045783 | No | - | 0.971985 | 0.044127 | No |
| B05CB | 0.98 (0.6-1.61) | 0.941993 | 0.042319 | No | 0.73 (0.39-1.38) | 0.327634 | 0.018223 | No |
| B05CX | 0.96 (0.24-3.87) | 0.95238 | 0.04262 | No | 0.55 (0.08-3.96) | 0.555875 | 0.026506 | No |
| B05XA | 1.21 (0.96-1.52) | 0.113986 | 0.009639 | No | 1.16 (0.88-1.53) | 0.281628 | 0.016265 | No |
| B05XB | - | 0.960994 | 0.043524 | No | - | 0.967274 | 0.041416 | No |
| B05XC | 1.08 (0.78-1.51) | 0.63805 | 0.032078 | No | 0.99 (0.67-1.45) | 0.95828 | 0.040211 | No |
| C01AA | 0.52 (0.17-1.61) | 0.253486 | 0.016114 | No | 0.49 (0.12-1.96) | 0.311085 | 0.01762 | No |
| C01BC | - | 0.968268 | 0.045934 | No | - | 0.972729 | 0.04488 | No |
| C01CA | 0.78 (0.55-1.11) | 0.162885 | 0.011898 | No | 0.79 (0.53-1.18) | 0.251373 | 0.014458 | No |
| C01DA | 1.42 (0.82-2.48) | 0.213044 | 0.014157 | No | 1.51 (0.8-2.84) | 0.206156 | 0.012349 | No |
| C01DX | 0.49 (0.07-3.5) | 0.478083 | 0.026054 | No | 0.73 (0.1-5.21) | 0.752453 | 0.033434 | No |
| C01EA | - | 0.970763 | 0.04744 | No | - | 0.973705 | 0.045783 | No |
| C01EB | 0.86 (0.53-1.39) | 0.540659 | 0.028765 | No | 0.8 (0.45-1.44) | 0.46245 | 0.02244 | No |
| C02CA | 1.13 (0.56-2.29) | 0.730462 | 0.034187 | No | 1.39 (0.65-2.96) | 0.394347 | 0.019729 | No |
| C02DB | 1.03 (0.51-2.08) | 0.935765 | 0.042018 | No | 0.85 (0.35-2.07) | 0.723811 | 0.03253 | No |
| C02DD | 2.53 (0.35-18.22) | 0.357593 | 0.020331 | No | - | 0.972594 | 0.044729 | No |
| C03AA | 0.61 (0.33-1.16) | 0.131225 | 0.010392 | No | 0.62 (0.29-1.32) | 0.213022 | 0.012952 | No |
| C03BA | 3.51 (1.3-9.48) | 0.013285 | 0.003614 | No | 2.57 (0.63-10.42) | 0.186358 | 0.011747 | No |
| C03CA | 1.24 (0.91-1.68) | 0.16628 | 0.012199 | No | 1.13 (0.79-1.61) | 0.518278 | 0.025 | No |
| C03DA | 1.19 (0.59-2.42) | 0.623337 | 0.031175 | No | 1.13 (0.5-2.55) | 0.774246 | 0.034036 | No |
| C03EA | - | 0.963814 | 0.044277 | No | - | 0.970657 | 0.043223 | No |
| C04AC | 1.74 (0.89-3.39) | 0.104792 | 0.009187 | No | 1.86 (0.82-4.2) | 0.136945 | 0.009488 | No |
| C04AD | - | 0.962284 | 0.043976 | No | - | 0.967945 | 0.041867 | No |
| C04AE | 0.81 (0.33-1.96) | 0.636387 | 0.031777 | No | 0.7 (0.22-2.18) | 0.534405 | 0.026205 | No |
| C04AF | 0.69 (0.22-2.17) | 0.527342 | 0.02756 | No | 0.93 (0.3-2.92) | 0.902676 | 0.038404 | No |
| C04AX | 0.75 (0.48-1.17) | 0.203901 | 0.013855 | No | 0.69 (0.41-1.17) | 0.164067 | 0.010542 | No |
| C05AD | 1.46 (0.8-2.67) | 0.218999 | 0.014608 | No | 1.51 (0.77-2.94) | 0.229955 | 0.013404 | No |
| C05BX | 0.6 (0.15-2.4) | 0.468367 | 0.025753 | No | 0.9 (0.22-3.65) | 0.887173 | 0.038102 | No |
| C05CA | 0.86 (0.56-1.31) | 0.47892 | 0.026205 | No | 1.08 (0.69-1.69) | 0.721986 | 0.032229 | No |
| C05CX | 0.75 (0.49-1.14) | 0.172931 | 0.0125 | No | 0.7 (0.42-1.17) | 0.171236 | 0.010994 | No |
| C07AA | 3.06 (1.44-6.51) | 0.003601 | 0.002259 | No | 3.68 (1.63-8.31) | 0.001757 | 0.001506 | No |
| C07AB | 1.04 (0.61-1.79) | 0.878621 | 0.040512 | No | 0.76 (0.38-1.55) | 0.456021 | 0.022139 | No |
| C07AG | 0.53 (0.28-1) | 0.048715 | 0.006928 | No | 0.51 (0.24-1.08) | 0.079789 | 0.007078 | No |
| C07CB | 2.6 (0.83-8.12) | 0.100186 | 0.008735 | No | 2.19 (0.54-8.8) | 0.271266 | 0.015663 | No |
| C08CA | 0.93 (0.66-1.3) | 0.662149 | 0.032681 | No | 1.01 (0.69-1.48) | 0.948796 | 0.03991 | No |
| C08DA | 0.48 (0.07-3.45) | 0.467932 | 0.025602 | No | - | 0.973506 | 0.045633 | No |
| C08DB | 0.45 (0.15-1.42) | 0.174326 | 0.012651 | No | 0.43 (0.11-1.72) | 0.232711 | 0.013554 | No |
| C09AA | 1.73 (0.89-3.36) | 0.107308 | 0.009488 | No | 1.37 (0.56-3.32) | 0.492149 | 0.023494 | No |
| C09BA | 5.94 (0.82-42.92) | 0.077287 | 0.007982 | No | 6.21 (0.86-45.05) | 0.071014 | 0.006777 | No |
| C09BB | 2.27 (0.32-16.26) | 0.413936 | 0.023494 | No | 2.68 (0.37-19.24) | 0.326204 | 0.018072 | No |
| C09CA | 0.87 (0.54-1.43) | 0.587667 | 0.029819 | No | 0.79 (0.43-1.44) | 0.437215 | 0.021084 | No |
| C09DA | 0.95 (0.52-1.74) | 0.864187 | 0.039759 | No | 1.18 (0.6-2.3) | 0.636079 | 0.02997 | No |
| C09DB | 2.11 (1.12-3.97) | 0.020171 | 0.004518 | No | 2.52 (1.29-4.91) | 0.006739 | 0.00241 | No |
| C10AA | 1.3 (0.85-1.98) | 0.233572 | 0.015211 | No | 1.05 (0.6-1.83) | 0.87475 | 0.0375 | No |
| C10AB | 0.91 (0.34-2.44) | 0.846183 | 0.039006 | No | 0.32 (0.05-2.27) | 0.253719 | 0.01491 | No |
| C10AC | 2.43 (0.34-17.43) | 0.375895 | 0.021687 | No | 4.11 (0.57-29.52) | 0.160271 | 0.010241 | No |
| C10AD | - | 0.969109 | 0.046687 | No | - | 0.975894 | 0.047289 | No |
| C10AX | 1.29 (0.18-9.22) | 0.798726 | 0.036747 | No | 1.89 (0.26-13.49) | 0.528057 | 0.026054 | No |
| C10BA | 1.47 (0.21-10.49) | 0.701471 | 0.033283 | No | - | 0.968621 | 0.042018 | No |
| C10BX | 1.2 (0.17-8.59) | 0.854287 | 0.039458 | No | 1.68 (0.24-12.05) | 0.604915 | 0.028464 | No |
| D01AC | 0.77 (0.38-1.56) | 0.472756 | 0.025904 | No | 1.07 (0.53-2.18) | 0.850339 | 0.036596 | No |
| D01AE | 0.59 (0.31-1.1) | 0.098313 | 0.008584 | No | 0.69 (0.35-1.34) | 0.272638 | 0.015813 | No |
| D01BA | 0.59 (0.19-1.84) | 0.362697 | 0.020934 | No | 0.79 (0.25-2.47) | 0.683681 | 0.031175 | No |
| D02AE | 0.32 (0.05-2.27) | 0.252751 | 0.015964 | No | 0.44 (0.06-3.13) | 0.410191 | 0.020331 | No |
| D03AX | 1.97 (0.27-14.16) | 0.500367 | 0.027259 | No | - | 0.976632 | 0.047741 | No |
| D06AX | 0.88 (0.55-1.42) | 0.599631 | 0.03012 | No | 0.86 (0.49-1.49) | 0.583111 | 0.027711 | No |
| D06BA | 4.19 (1.02-17.24) | 0.047153 | 0.006777 | No | - | 0.96627 | 0.041114 | No |
| D06BB | 0.66 (0.21-2.07) | 0.479673 | 0.026355 | No | - | 0.971564 | 0.043825 | No |
| D07AA | 1.36 (0.77-2.43) | 0.293725 | 0.017018 | No | 1.49 (0.77-2.9) | 0.241126 | 0.014006 | No |
| D07AB | 0.93 (0.55-1.59) | 0.795474 | 0.036596 | No | 0.95 (0.5-1.79) | 0.869092 | 0.037349 | No |
| D07AC | 1.03 (0.71-1.5) | 0.871917 | 0.040211 | No | 1.04 (0.68-1.6) | 0.843673 | 0.035994 | No |
| D07AD | 0.7 (0.39-1.28) | 0.248542 | 0.015813 | No | 0.64 (0.3-1.35) | 0.241161 | 0.014157 | No |
| D07CC | 1.33 (0.76-2.32) | 0.314964 | 0.018072 | No | 1.29 (0.66-2.5) | 0.46189 | 0.022289 | No |
| D08AG | 1 (0.41-2.43) | 0.997485 | 0.049849 | No | 0.92 (0.34-2.5) | 0.876169 | 0.037651 | No |
| D11AH | 0.71 (0.1-5.06) | 0.731215 | 0.034337 | No | 1.11 (0.16-7.96) | 0.915492 | 0.039006 | No |
| D11AX | 1.93 (0.48-7.8) | 0.358166 | 0.020482 | No | - | 0.970079 | 0.042771 | No |
| G01AA | 1.97 (0.61-6.32) | 0.253938 | 0.016265 | No | 0.6 (0.08-4.37) | 0.614498 | 0.028614 | No |
| G01AF | 1.44 (0.63-3.27) | 0.390985 | 0.02244 | No | 1.28 (0.52-3.18) | 0.589871 | 0.028012 | No |
| G01AX | 0.69 (0.17-2.79) | 0.601188 | 0.030271 | No | 0.79 (0.19-3.2) | 0.734962 | 0.032831 | No |
| G02AB | - | 0.969711 | 0.047139 | No | - | 0.972954 | 0.045181 | No |
| G03BA | - | 0.969027 | 0.046536 | No | - | 0.974075 | 0.046687 | No |
| G03CA | 1.03 (0.5-2.12) | 0.926355 | 0.041717 | No | 0.92 (0.4-2.09) | 0.832654 | 0.035843 | No |
| G03CX | - | 0.960805 | 0.043373 | No | - | 0.966796 | 0.041265 | No |
| G03DA | 56.16 (7.29-432.47) | 0.00011 | 0.000602 | Yes | 80.41 (9.81-659.21) | 0.000044 | 0.000301 | Yes |
| G03FA | - | 0.972385 | 0.047892 | No | - | 0.976299 | 0.04759 | No |
| G03HB | - | 0.976726 | 0.048494 | No | - | 0.980435 | 0.048494 | No |
| G04BD | 1.32 (0.83-2.11) | 0.241053 | 0.015512 | No | 1.36 (0.8-2.31) | 0.250279 | 0.014307 | No |
| G04BX | 1.39 (0.2-9.92) | 0.743022 | 0.03494 | No | 1.9 (0.27-13.6) | 0.522834 | 0.025602 | No |
| G04CA | 1.22 (0.81-1.84) | 0.332157 | 0.018976 | No | 1 (0.59-1.69) | 0.997202 | 0.05 | No |
| G04CB | 0.89 (0.46-1.75) | 0.736112 | 0.034639 | No | 1.01 (0.44-2.3) | 0.983679 | 0.049247 | No |
| G04CX | 1.73 (0.43-6.97) | 0.439693 | 0.024548 | No | 2.76 (0.68-11.18) | 0.154083 | 0.01009 | No |
| H01BA | 1.32 (0.19-9.46) | 0.779642 | 0.035994 | No | - | 0.967443 | 0.041566 | No |
| H01BB | - | 0.973152 | 0.048193 | No | - | 0.976206 | 0.04744 | No |
| H01CB | 2.3 (0.73-7.24) | 0.155996 | 0.011747 | No | 1.59 (0.39-6.46) | 0.516426 | 0.024699 | No |
| H02AB | 0.8 (0.62-1.05) | 0.104664 | 0.009036 | No | 0.72 (0.53-0.99) | 0.040743 | 0.00512 | No |
| H02BX | 1.73 (0.71-4.18) | 0.226525 | 0.014759 | No | 0.89 (0.22-3.57) | 0.86643 | 0.037048 | No |
| H03AA | 0.39 (0.06-2.81) | 0.352427 | 0.02003 | No | 0.53 (0.07-3.77) | 0.52272 | 0.025452 | No |
| H03BB | 1.05 (0.15-7.48) | 0.96504 | 0.045181 | No | 1.33 (0.19-9.56) | 0.775363 | 0.034187 | No |
| H03CA | 2.02 (0.28-14.5) | 0.484399 | 0.026958 | No | 2.3 (0.32-16.54) | 0.40777 | 0.020181 | No |
| H04AA | 18.79 (2.56-137.75) | 0.003905 | 0.00241 | No | 23.76 (3.19-176.91) | 0.001982 | 0.001657 | No |
| H05BA | 0.42 (0.06-3.01) | 0.388438 | 0.022289 | No | - | 0.966222 | 0.040964 | No |
| J01AA | 0.51 (0.21-1.23) | 0.132831 | 0.010542 | No | 0.27 (0.07-1.07) | 0.06256 | 0.006024 | No |
| J01BA | - | 0.95828 | 0.043072 | No | - | 0.965876 | 0.040813 | No |
| J01CA | 0.62 (0.4-0.98) | 0.039345 | 0.005873 | No | 0.75 (0.45-1.24) | 0.26475 | 0.015361 | No |
| J01CE | - | 0.961938 | 0.043675 | No | - | 0.967521 | 0.041717 | No |
| J01CR | 0.96 (0.72-1.27) | 0.768773 | 0.035392 | No | 0.89 (0.64-1.24) | 0.490107 | 0.023193 | No |
| J01DB | 0.83 (0.62-1.12) | 0.231095 | 0.01506 | No | 0.71 (0.49-1.03) | 0.070507 | 0.006627 | No |
| J01DC | 0.93 (0.72-1.19) | 0.559541 | 0.029066 | No | 0.73 (0.53-0.98) | 0.039194 | 0.004819 | No |
| J01DD | 1.14 (0.86-1.52) | 0.364259 | 0.021084 | No | 0.97 (0.68-1.37) | 0.846766 | 0.036446 | No |
| J01DE | - | 0.963935 | 0.044578 | No | - | 0.96907 | 0.042169 | No |
| J01DH | 0.21 (0.03-1.53) | 0.124028 | 0.01009 | No | - | 0.971075 | 0.043675 | No |
| J01EE | 0.71 (0.34-1.51) | 0.375478 | 0.021536 | No | 0.7 (0.29-1.71) | 0.437605 | 0.021235 | No |
| J01FA | 0.97 (0.7-1.34) | 0.830708 | 0.038253 | No | 0.85 (0.57-1.27) | 0.43507 | 0.020934 | No |
| J01FF | 0.81 (0.51-1.29) | 0.366416 | 0.021235 | No | 0.84 (0.49-1.44) | 0.52671 | 0.025904 | No |
| J01GB | 0.9 (0.69-1.16) | 0.413911 | 0.023343 | No | 0.79 (0.57-1.07) | 0.128952 | 0.008886 | No |
| J01MA | 0.97 (0.76-1.24) | 0.80212 | 0.037048 | No | 1.03 (0.78-1.37) | 0.846431 | 0.036295 | No |
| J01RA | 0.45 (0.06-3.22) | 0.428092 | 0.024096 | No | 0.59 (0.08-4.22) | 0.598848 | 0.028163 | No |
| J01XA | 0.63 (0.15-2.54) | 0.513044 | 0.02741 | No | 0.88 (0.22-3.57) | 0.852101 | 0.036747 | No |
| J01XC | - | 0.969687 | 0.046988 | No | - | 0.97396 | 0.046386 | No |
| J01XD | 0.59 (0.43-0.81) | 0.001078 | 0.001355 | No | 0.5 (0.35-0.73) | 0.000317 | 0.001054 | No |
| J02AB | 12.64 (1.74-91.54) | 0.012063 | 0.003313 | No | 11.74 (1.61-85.54) | 0.015046 | 0.003614 | No |
| J02AC | 0.86 (0.52-1.42) | 0.554438 | 0.028916 | No | 1.01 (0.58-1.76) | 0.983242 | 0.049096 | No |
| J04AB | 2.09 (0.66-6.55) | 0.208127 | 0.014006 | No | 1.99 (0.49-8.06) | 0.335194 | 0.018675 | No |
| J04AC | 1.43 (0.35-5.78) | 0.615566 | 0.030723 | No | 1.01 (0.14-7.22) | 0.995553 | 0.049699 | No |
| J04AK | 1.43 (0.35-5.78) | 0.615789 | 0.030873 | No | 1.01 (0.14-7.21) | 0.995681 | 0.049849 | No |
| J05AB | 0.41 (0.17-1) | 0.049963 | 0.007078 | No | 0.12 (0.02-0.82) | 0.031138 | 0.004066 | No |
| J05AF | 14.93 (2.04-109.42) | 0.007806 | 0.003012 | No | - | 0.978029 | 0.048193 | No |
| J05AH | - | 0.970749 | 0.047289 | No | - | 0.973715 | 0.045934 | No |
| J05AP | - | 0.963733 | 0.044127 | No | - | 0.970095 | 0.042922 | No |
| J05AX | 0.82 (0.12-5.83) | 0.841141 | 0.038554 | No | - | 0.975416 | 0.046988 | No |
| J06BB | 0.69 (0.17-2.76) | 0.59681 | 0.02997 | No | - | 0.965386 | 0.040512 | No |
| M01AB | 0.94 (0.73-1.21) | 0.62966 | 0.031476 | No | 0.93 (0.69-1.25) | 0.621395 | 0.029217 | No |
| M01AC | 1.14 (0.84-1.56) | 0.400767 | 0.022892 | No | 1.13 (0.78-1.64) | 0.508303 | 0.024247 | No |
| M01AE | 0.67 (0.52-0.86) | 0.002207 | 0.002108 | No | 0.58 (0.43-0.8) | 0.000725 | 0.001205 | No |
| M01AG | 0.95 (0.62-1.47) | 0.820499 | 0.037801 | No | 1.06 (0.65-1.72) | 0.816807 | 0.035693 | No |
| M01AH | 1.95 (1.03-3.68) | 0.040765 | 0.006175 | No | 1.6 (0.71-3.63) | 0.260773 | 0.015211 | No |
| M01AX | 1.05 (0.8-1.37) | 0.740843 | 0.034789 | No | 0.95 (0.69-1.32) | 0.770485 | 0.033886 | No |
| M02AA | 0.87 (0.56-1.35) | 0.530508 | 0.028012 | No | 0.74 (0.43-1.28) | 0.277662 | 0.016114 | No |
| M03AB | 1.02 (0.52-2) | 0.945055 | 0.04247 | No | 1.03 (0.51-2.1) | 0.930104 | 0.039608 | No |
| M03AC | 0.68 (0.48-0.97) | 0.030983 | 0.005422 | No | 0.57 (0.38-0.87) | 0.008353 | 0.00256 | No |
| M03BA | 1.29 (0.79-2.1) | 0.311965 | 0.017771 | No | 1.04 (0.55-1.96) | 0.907615 | 0.038554 | No |
| M03BC | 0.76 (0.44-1.3) | 0.314755 | 0.017922 | No | 0.76 (0.4-1.42) | 0.385231 | 0.019428 | No |
| M03BX | 1.12 (0.85-1.47) | 0.431798 | 0.024398 | No | 1.18 (0.85-1.62) | 0.324416 | 0.017922 | No |
| M03CA | 0.9 (0.22-3.61) | 0.880639 | 0.040813 | No | 0.64 (0.09-4.53) | 0.650959 | 0.030422 | No |
| M04AA | 4.96 (2.33-10.55) | 0.000032 | 0.000452 | No | 4.48 (1.84-10.91) | 0.000982 | 0.001355 | No |
| M04AC | 2.59 (0.64-10.44) | 0.181011 | 0.012801 | No | 1.8 (0.25-12.9) | 0.557564 | 0.026807 | No |
| M05BA | 0.99 (0.5-1.96) | 0.982756 | 0.049247 | No | 0.79 (0.32-1.94) | 0.603146 | 0.028313 | No |
| M05BB | 1.68 (0.24-12.07) | 0.604371 | 0.030572 | No | 2.74 (0.38-19.73) | 0.31614 | 0.017771 | No |
| M05BX | - | 0.964358 | 0.044729 | No | - | 0.969376 | 0.042319 | No |
| M09AB | 0.73 (0.56-0.95) | 0.017705 | 0.004066 | No | 0.72 (0.53-0.97) | 0.033679 | 0.004518 | No |
| M09AX | 0.87 (0.56-1.36) | 0.538915 | 0.028464 | No | 0.99 (0.61-1.62) | 0.977069 | 0.047892 | No |
| N01AB | 0.67 (0.47-0.95) | 0.026696 | 0.00497 | No | 0.58 (0.38-0.88) | 0.010307 | 0.002861 | No |
| N01AF | 0.4 (0.1-1.63) | 0.203285 | 0.013554 | No | 0.48 (0.12-1.95) | 0.307116 | 0.017018 | No |
| N01AH | 0.54 (0.38-0.77) | 0.000607 | 0.000753 | Yes | 0.44 (0.29-0.67) | 0.000117 | 0.000904 | Yes |
| N01AX | 0.83 (0.56-1.23) | 0.342068 | 0.019428 | No | 0.74 (0.47-1.17) | 0.195395 | 0.011898 | No |
| N01BB | 0.98 (0.77-1.25) | 0.868817 | 0.04006 | No | 0.83 (0.62-1.11) | 0.210114 | 0.012651 | No |
| N02AA | 1.55 (1.02-2.36) | 0.042495 | 0.006476 | No | 1.18 (0.7-1.97) | 0.536399 | 0.026355 | No |
| N02AB | 0.82 (0.66-1.02) | 0.074158 | 0.007831 | No | 0.82 (0.64-1.06) | 0.132408 | 0.009036 | No |
| N02AE | 2.82 (0.39-20.36) | 0.305182 | 0.01762 | No | 4.56 (0.63-33.23) | 0.134069 | 0.009337 | No |
| N02AF | 0.42 (0.15-1.12) | 0.08271 | 0.008283 | No | 0.13 (0.02-0.94) | 0.042963 | 0.005271 | No |
| N02AJ | 1.34 (1.01-1.77) | 0.039656 | 0.006024 | No | 1.31 (0.95-1.81) | 0.097142 | 0.00738 | No |
| N02AX | 1.05 (0.82-1.35) | 0.708514 | 0.033584 | No | 1.08 (0.81-1.44) | 0.617083 | 0.028765 | No |
| N02BA | 0.88 (0.44-1.79) | 0.731319 | 0.034488 | No | 1.01 (0.45-2.28) | 0.977136 | 0.048042 | No |
| N02BE | 0.8 (0.58-1.1) | 0.165428 | 0.012048 | No | 0.74 (0.51-1.09) | 0.127265 | 0.008735 | No |
| N03AA | 2.29 (0.73-7.18) | 0.155003 | 0.011596 | No | 2.69 (0.67-10.92) | 0.165142 | 0.010693 | No |
| N03AE | 0.87 (0.28-2.72) | 0.808052 | 0.037349 | No | 0.38 (0.05-2.7) | 0.331735 | 0.018524 | No |
| N03AF | 0.99 (0.25-3.97) | 0.984212 | 0.049398 | No | 0.62 (0.09-4.39) | 0.627782 | 0.029669 | No |
| N03AG | 2.04 (0.84-4.94) | 0.11574 | 0.00994 | No | 1.22 (0.3-4.91) | 0.784156 | 0.034639 | No |
| N03AX | 1.26 (0.79-2.02) | 0.335244 | 0.019127 | No | 1.16 (0.65-2.09) | 0.619876 | 0.028916 | No |
| N04AC | - | 0.967548 | 0.045633 | No | - | 0.973433 | 0.045482 | No |
| N04BA | - | 0.964655 | 0.04488 | No | - | 0.970624 | 0.043072 | No |
| N04BC | - | 0.968571 | 0.046235 | No | - | 0.972984 | 0.045331 | No |
| N05AA | - | 0.967205 | 0.045482 | No | - | 0.981416 | 0.048645 | No |
| N05AB | 0.86 (0.21-3.44) | 0.825459 | 0.038102 | No | 1.26 (0.31-5.08) | 0.743998 | 0.032982 | No |
| N05AD | 1.77 (0.57-5.52) | 0.328168 | 0.018675 | No | 2.47 (0.79-7.74) | 0.120145 | 0.008584 | No |
| N05AH | 1.71 (0.42-6.89) | 0.452422 | 0.025 | No | 1.14 (0.16-8.17) | 0.895793 | 0.038253 | No |
| N05AX | 3.98 (0.55-28.8) | 0.171979 | 0.012349 | No | - | 0.969932 | 0.04262 | No |
| N05BA | 0.99 (0.77-1.27) | 0.916351 | 0.041114 | No | 0.99 (0.74-1.32) | 0.949889 | 0.04006 | No |
| N05BB | 0.73 (0.49-1.1) | 0.13777 | 0.010994 | No | 0.86 (0.55-1.35) | 0.518019 | 0.024849 | No |
| N05BE | 0.4 (0.17-0.97) | 0.042432 | 0.006325 | No | 0.35 (0.11-1.1) | 0.071742 | 0.006928 | No |
| N05CD | 0.79 (0.64-0.99) | 0.038043 | 0.005723 | No | 0.81 (0.63-1.05) | 0.110168 | 0.007681 | No |
| N05CF | 1.18 (0.76-1.82) | 0.467866 | 0.025452 | No | 1.36 (0.84-2.21) | 0.208774 | 0.0125 | No |
| N06AA | 1.28 (0.81-2.04) | 0.293505 | 0.016867 | No | 1.5 (0.9-2.49) | 0.119596 | 0.008434 | No |
| N06AB | 1.92 (0.98-3.75) | 0.055797 | 0.007229 | No | 2.15 (1.01-4.6) | 0.047449 | 0.005572 | No |
| N06AX | 1.56 (0.91-2.68) | 0.104898 | 0.009337 | No | 1.28 (0.63-2.6) | 0.496715 | 0.023795 | No |
| N06BX | 1.12 (0.53-2.37) | 0.770485 | 0.035542 | No | 0.47 (0.12-1.91) | 0.293503 | 0.016867 | No |
| N06DA | 2.29 (0.85-6.18) | 0.103523 | 0.008886 | No | 2.35 (0.74-7.43) | 0.145144 | 0.009639 | No |
| N06DX | 0.78 (0.49-1.25) | 0.299763 | 0.017319 | No | 0.89 (0.53-1.51) | 0.677585 | 0.031024 | No |
| N07AA | 0.47 (0.33-0.66) | 0.00002 | 0.000301 | Yes | 0.42 (0.28-0.62) | 0.000019 | 0.000151 | Yes |
| N07AB | 0.71 (0.18-2.88) | 0.636546 | 0.031928 | No | 0.46 (0.06-3.29) | 0.439386 | 0.021386 | No |
| N07AX | 1.41 (0.63-3.17) | 0.409656 | 0.023193 | No | 1.74 (0.72-4.25) | 0.221956 | 0.013102 | No |
| N07CA | 1.6 (0.6-4.31) | 0.350457 | 0.01988 | No | 0.61 (0.09-4.39) | 0.626214 | 0.029518 | No |
| N07XX | - | 0.97303 | 0.048042 | No | - | 0.97585 | 0.047139 | No |
| P01AB | 0.47 (0.15-1.47) | 0.194406 | 0.013404 | No | 0.68 (0.22-2.12) | 0.505433 | 0.024096 | No |
| P01BA | - | 0.971711 | 0.047741 | No | - | 0.974013 | 0.046536 | No |
| P02BA | - | 0.979874 | 0.049096 | No | - | 0.983715 | 0.049398 | No |
| R01AD | 1.22 (0.5-2.97) | 0.656132 | 0.03238 | No | 1.12 (0.36-3.51) | 0.844259 | 0.036145 | No |
| R01AX | 4.89 (1.78-13.44) | 0.00212 | 0.001958 | No | 7.49 (2.7-20.8) | 0.000111 | 0.000753 | No |
| R01BA | 1.04 (0.78-1.38) | 0.795458 | 0.036446 | No | 1 (0.72-1.4) | 0.987363 | 0.049548 | No |
| R02AA | 0.68 (0.36-1.28) | 0.233725 | 0.015361 | No | 0.33 (0.12-0.88) | 0.026684 | 0.003916 | No |
| R02AD | 0.99 (0.76-1.29) | 0.958705 | 0.043223 | No | 0.86 (0.62-1.18) | 0.347847 | 0.018825 | No |
| R03AC | 0.69 (0.36-1.34) | 0.276027 | 0.016416 | No | 0.51 (0.21-1.23) | 0.133546 | 0.009187 | No |
| R03AK | 0.98 (0.4-2.36) | 0.955914 | 0.042922 | No | 0.54 (0.13-2.16) | 0.380328 | 0.019277 | No |
| R03BA | 0.54 (0.08-3.81) | 0.533405 | 0.028313 | No | 0.77 (0.11-5.46) | 0.78913 | 0.03494 | No |
| R03BB | 1.27 (0.65-2.49) | 0.480099 | 0.026506 | No | 0.94 (0.38-2.29) | 0.883037 | 0.037801 | No |
| R03CA | 0.48 (0.31-0.76) | 0.001764 | 0.001807 | No | 0.45 (0.27-0.76) | 0.002555 | 0.001958 | No |
| R03CC | 1.05 (0.73-1.51) | 0.801924 | 0.036898 | No | 1.07 (0.7-1.65) | 0.752394 | 0.033283 | No |
| R03CK | 1.76 (0.44-7.09) | 0.427083 | 0.023946 | No | 3.11 (0.77-12.57) | 0.111459 | 0.007831 | No |
| R03DA | 1.07 (0.74-1.55) | 0.722497 | 0.034036 | No | 0.87 (0.54-1.39) | 0.555979 | 0.026657 | No |
| R03DC | 1.05 (0.57-1.91) | 0.878006 | 0.040361 | No | 0.9 (0.43-1.92) | 0.793737 | 0.03509 | No |
| R03DX | - | 0.979029 | 0.048645 | No | - | 0.983106 | 0.048946 | No |
| R05CA | 0.6 (0.4-0.92) | 0.019699 | 0.004217 | No | 0.56 (0.33-0.95) | 0.031406 | 0.004217 | No |
| R05CB | 0.85 (0.66-1.1) | 0.216764 | 0.014307 | No | 0.75 (0.56-1) | 0.04968 | 0.005723 | No |
| R05DA | 1.1 (0.45-2.66) | 0.838748 | 0.038404 | No | 1.43 (0.59-3.46) | 0.433389 | 0.020783 | No |
| R05DB | 0.87 (0.63-1.19) | 0.375981 | 0.021837 | No | 0.95 (0.66-1.35) | 0.75809 | 0.033735 | No |
| R05FA | 1.01 (0.78-1.32) | 0.926207 | 0.041566 | No | 0.97 (0.71-1.33) | 0.859199 | 0.036898 | No |
| R06AA | 0.89 (0.64-1.23) | 0.467248 | 0.025301 | No | 0.91 (0.62-1.35) | 0.648378 | 0.03012 | No |
| R06AB | 1.13 (0.89-1.45) | 0.322164 | 0.018373 | No | 1.1 (0.82-1.48) | 0.51228 | 0.024548 | No |
| R06AD | 0.78 (0.46-1.31) | 0.345496 | 0.019578 | No | 0.91 (0.51-1.63) | 0.757233 | 0.033584 | No |
| R06AE | 1.02 (0.73-1.42) | 0.921402 | 0.041265 | No | 1.21 (0.84-1.76) | 0.308218 | 0.017169 | No |
| R06AX | 1 (0.75-1.32) | 0.989858 | 0.049699 | No | 0.96 (0.68-1.34) | 0.807392 | 0.035241 | No |
| R07AB | - | 0.963872 | 0.044428 | No | - | 0.971014 | 0.043524 | No |
| R07AX | 1.53 (0.85-2.75) | 0.152752 | 0.011295 | No | 1.3 (0.61-2.78) | 0.503224 | 0.023946 | No |
| S01AA | 0.69 (0.39-1.19) | 0.181272 | 0.012952 | No | 0.65 (0.34-1.27) | 0.21051 | 0.012801 | No |
| S01AD | - | 0.968964 | 0.046386 | No | - | 0.97379 | 0.046084 | No |
| S01AE | 1.05 (0.73-1.5) | 0.792393 | 0.036295 | No | 1.06 (0.7-1.61) | 0.777839 | 0.034337 | No |
| S01BA | 0.83 (0.56-1.21) | 0.323707 | 0.018524 | No | 0.78 (0.49-1.23) | 0.283349 | 0.016416 | No |
| S01BC | 0.6 (0.19-1.87) | 0.380031 | 0.021988 | No | 0.91 (0.29-2.84) | 0.86673 | 0.037199 | No |
| S01CA | 0.73 (0.38-1.42) | 0.35889 | 0.020633 | No | 0.71 (0.32-1.6) | 0.412636 | 0.020633 | No |
| S01EA | - | 0.97568 | 0.048343 | No | - | 0.979598 | 0.048343 | No |
| S01EB | 1.02 (0.14-7.26) | 0.987158 | 0.049548 | No | 1.64 (0.23-11.76) | 0.620275 | 0.029066 | No |
| S01EC | 0.56 (0.14-2.24) | 0.407591 | 0.023042 | No | 0.43 (0.06-3.06) | 0.397281 | 0.01988 | No |
| S01ED | 0.75 (0.24-2.35) | 0.620065 | 0.031024 | No | 0.39 (0.06-2.82) | 0.35248 | 0.018976 | No |
| S01EE | 1.57 (0.58-4.23) | 0.370108 | 0.021386 | No | 1.18 (0.29-4.78) | 0.815589 | 0.035542 | No |
| S01FA | 0.91 (0.29-2.84) | 0.86867 | 0.03991 | No | - | 0.970932 | 0.043373 | No |
| S01GX | 0.98 (0.49-1.98) | 0.961994 | 0.043825 | No | 1.16 (0.55-2.47) | 0.693819 | 0.031627 | No |
| S01HA | 1.08 (0.45-2.63) | 0.859415 | 0.039608 | No | 1.26 (0.47-3.39) | 0.650386 | 0.030271 | No |
| S01JA | 3.6 (1.15-11.3) | 0.028005 | 0.00512 | No | 3.08 (0.76-12.46) | 0.114871 | 0.008133 | No |
| S01KA | 1.84 (0.68-4.94) | 0.228661 | 0.01491 | No | 1.99 (0.64-6.24) | 0.237626 | 0.013705 | No |
| S01KX | - | 0.979504 | 0.048795 | No | - | 0.982865 | 0.048795 | No |
| S01XA | 0.88 (0.62-1.25) | 0.48025 | 0.026657 | No | 0.93 (0.62-1.41) | 0.745176 | 0.033133 | No |
| S02AA | 1.11 (0.41-2.96) | 0.843491 | 0.038705 | No | 1.52 (0.57-4.09) | 0.405817 | 0.02003 | No |
| S02CA | 3.45 (0.48-24.87) | 0.218671 | 0.014458 | No | 3.89 (0.54-28.1) | 0.17836 | 0.011596 | No |
| S03AA | - | 0.969491 | 0.046837 | No | - | 0.97461 | 0.046837 | No |
| V03AB | 0.49 (0.18-1.31) | 0.154759 | 0.011446 | No | 0.51 (0.16-1.61) | 0.252637 | 0.014759 | No |
| V03AE | 4.05 (1.5-10.92) | 0.005756 | 0.00256 | No | 4.18 (1.33-13.16) | 0.014391 | 0.003313 | No |
| V03AF | 0.78 (0.47-1.3) | 0.338117 | 0.019277 | No | 0.73 (0.41-1.29) | 0.275492 | 0.015964 | No |
| V04CA | 1.77 (0.44-7.15) | 0.422883 | 0.023795 | No | 2.64 (0.65-10.72) | 0.1751 | 0.011446 | No |
| V04CH | 1.28 (0.18-9.11) | 0.807547 | 0.037199 | No | 1.64 (0.23-11.73) | 0.623203 | 0.029367 | No |
| V04CX | 2.52 (0.62-10.2) | 0.194174 | 0.013253 | No | 2.83 (0.7-11.5) | 0.145451 | 0.009789 | No |
| V06DB | 2 (0.5-8.06) | 0.33158 | 0.018825 | No | 1.12 (0.16-8.03) | 0.908845 | 0.038705 | No |
| V06DX | 10.98 (2.71-44.49) | 0.000785 | 0.000904 | No | - | 0.972393 | 0.044428 | No |
| V07AB | 1.05 (0.76-1.45) | 0.768047 | 0.035241 | No | 0.92 (0.63-1.34) | 0.654754 | 0.030572 | No |
| V08AA | 0.68 (0.47-0.99) | 0.044397 | 0.006627 | No | 0.8 (0.53-1.21) | 0.289518 | 0.016717 | No |
| V08AB | 0.89 (0.69-1.14) | 0.35375 | 0.020181 | No | 0.93 (0.68-1.26) | 0.630903 | 0.029819 | No |
| V08BA | 0.87 (0.66-1.13) | 0.295112 | 0.017169 | No | 0.76 (0.55-1.06) | 0.105725 | 0.00753 | No |
| V08CA | 1.38 (1.06-1.79) | 0.017312 | 0.003916 | No | 1.38 (1.03-1.86) | 0.033272 | 0.004367 | No |
| V08CB | 24.37 (5.8-102.41) | 0.000013 | 0.000151 | No | 19.73 (4.65-83.68) | 0.000052 | 0.000452 | No |
| V09BA | 1.08 (0.65-1.79) | 0.774366 | 0.035693 | No | 0.81 (0.43-1.53) | 0.511473 | 0.024398 | No |
| V09CA | 3.51 (0.86-14.34) | 0.079919 | 0.008133 | No | 2.93 (0.41-21.15) | 0.287439 | 0.016566 | No |
| V09FX | 1.33 (0.92-1.91) | 0.125943 | 0.010241 | No | 0.92 (0.57-1.47) | 0.71381 | 0.031928 | No |
| V09GA | 1.54 (0.57-4.13) | 0.395508 | 0.02259 | No | 1.11 (0.28-4.48) | 0.883637 | 0.037952 | No |
| V09GX | 0.57 (0.27-1.21) | 0.14196 | 0.011145 | No | 0.56 (0.23-1.37) | 0.205664 | 0.012048 | No |
| V09IX | 1.39 (1.09-1.78) | 0.007885 | 0.003163 | No | 1.51 (1.14-2) | 0.00382 | 0.002108 | No |

Supplementary Table 3. The Anatomical Therapeutic Chemical (ATC) level 2 classification codes for the drugs included in the analysis.

| ATC code | Drug class | ATC code | Drug class |
| --- | --- | --- | --- |
| A01 | Stomatological preparations | H03 | Thyroid therapy |
| A02 | Drugs for acid related disorders | H04 | Pancreatic hormones |
| A03 | Drugs for functional gastrointestinal disorders | H05 | Calcium homeostasis |
| A04 | Antiemetics and antinauseants | J01 | Antibacterials for systemic use |
| A05 | Bile and liver therapy | J02 | Antimycotics for systemic use |
| A06 | Drugs for constipation | J04 | Antimycobacterials |
| A07 | Antidiarrheals, intestinal anti-inflammatory/anti-infective agents | J05 | Antivirals for systemic use |
| A09 | Digestives, including enzymes | J06 | Immune sera and immunoglobulins |
| A10 | Drugs used for diabetes | M01 | Anti-inflammatory and antirheumatic products |
| A11 | Vitamins | M02 | Topical products for joint and muscular pain |
| A12 | Mineral supplements | M03 | Muscle relaxants |
| A16 | Other alimentary tract and metabolism products | M04 | Antigout preparations |
| B01 | Antithrombotic agents | M05 | Drugs for the treatment of bone diseases |
| B02 | Antihemorrhagics | M09 | Other drugs for disorders of the musculoskeletal system |
| B03 | Antianemic preparations | N01 | Anesthetics |
| B05 | Blood substitutes and perfusion solutions | N02 | Analgesics |
| C01 | Cardiac therapy | N03 | Antiepileptics |
| C02 | Antihypertensives | N04 | Anti-Parkinson drugs |
| C03 | Diuretics | N05 | Psycholeptics |
| C04 | Peripheral vasodilators | N06 | Psychoanaleptics |
| C05 | Vasoprotectives | N07 | Other nervous system drugs |
| C07 | Beta-blocking agents | P01 | Antiprotozoals |
| C08 | Calcium channel blockers | P02 | Anthelmintics |
| C09 | Agents acting on the renin–angiotensin system | R01 | Nasal preparations |
| C10 | Lipid modifying agents | R02 | Throat preparations |
| D01 | Antifungals for dermatological use | R03 | Drugs for obstructive airway diseases |
| D02 | Emollients and protectives | R05 | Cough and cold preparations |
| D03 | Preparations for the treatment of wounds and ulcers | R06 | Antihistamines for systemic use |
| D06 | Antibiotics and chemotherapeutics for dermatological use | R07 | Other respiratory system products |
| D07 | Corticosteroids, dermatological preparations | S01 | Ophthalmologicals |
| D08 | Antiseptics and disinfectants | S02 | Otologicals |
| D11 | Other dermatological preparations | S03 | Ophthalmological and otological preparations |
| G01 | Gynecological anti-infectives and antiseptics | V03 | All other therapeutic products |
| G02 | Other gynecologicals | V04 | Diagnostic agents |
| G03 | Sex hormones and modulators of the genital system | V06 | General nutrients |
| G04 | Urologicals | V07 | All other nontherapeutic products |
| H01 | Pituitary and hypothalamic hormones and analogs | V08 | Contrast media |
| H02 | Corticosteroids for systemic use | V09 | Diagnostic radiopharmaceuticals |

Supplementary Table 4. The Anatomical Therapeutic Chemical (ATC) level 4 classification codes for drugs included in the analysis.

| ATC code | Drug class | | ATC code | Drug class |
| --- | --- | --- | --- | --- |
| A01AC | | Corticosteroids for local oral treatment | A16AA | Amino acids and derivatives |
| A01AD | | Other agents for local oral treatment | A16AX | Various alimentary tract and metabolism products |
| A02AA | | Magnesium compounds | B01AA | Vitamin K antagonists |
| A02AB | | Aluminum compounds | B01AB | Heparin group |
| A02AC | | Calcium compounds | B01AC | Platelet aggregation inhibitors excluding heparin |
| A02AD | | Combinations and complexes of aluminum, calcium and magnesium compounds | B01AX | Other antithrombotic agents |
| A02BA | | H2-receptor antagonists | B02AA | Amino acids |
| A02BB | | Prostaglandins | B02AB | Proteinase inhibitors |
| A02BC | | Proton pump inhibitors | B02BA | Vitamin K |
| A02BX | | Other drugs for peptic ulcers and gastro-esophageal reflux disease (GERD) | B02BC | Local hemostatics |
| A03AA | | Synthetic anticholinergics, esters with tertiary amino group | B02BX | Other systemic hemostatics |
| A03AB | | Synthetic anticholinergics, quaternary ammonium compounds | B03AA | Iron bivalent, oral preparations |
| A03AC | | Synthetic antispasmodics, amides with tertiary amines | B03AB | Iron trivalent, oral preparations |
| A03AD | | Papaverine and derivatives | B03AC | Iron, parenteral preparations |
| A03AE | | Serotonin receptor antagonists | B03AD | Iron in combination with folic acid |
| A03AX | | Other drugs for functional gastrointestinal disorders | B03BA | Vitamin B12 (cyanocobalamin and analogs) |
| A03BA | | Belladonna alkaloids, tertiary amines | B03BB | Folic acid and derivatives |
| A03BB | | Belladonna alkaloids, semisynthetic, quaternary ammonium compounds | B03XA | Other antianemic preparations |
| A03CA | | Synthetic anticholinergic agents in combination with psycholeptics | B05AA | Blood substitutes and plasma protein fractions |
| A03FA | | Propulsives | B05BA | Solutions for parenteral nutrition |
| A04AA | | Serotonin (5-HT3) antagonists | B05BB | Solutions affecting the electrolyte balance |
| A04AD | | Other antiemetics | B05BC | Solutions causing osmotic diuresis |
| A05AA | | Bile acids and derivatives | B05CA | Anti-infectives |
| A05AX | | Other drugs for bile therapy | B05CB | Salt solutions |
| A05BA | | Liver therapy | B05CX | Other irrigating solutions |
| A06AB | | Contact laxatives | B05XA | Electrolyte solutions |
| A06AC | | Bulk-forming laxatives | B05XB | Amino acids |
| A06AD | | Osmotically acting laxatives | B05XC | Vitamins |
| A06AG | | Enemas | C01AA | Digitalis glycosides |
| A07AA | | Antibiotics | C01BC | Antiarrhythmics, class Ic |
| A07AX | | Other intestinal anti-infectives | C01CA | Adrenergic and dopaminergic agents |
| A07BC | | Other intestinal adsorbents | C01DA | Organic nitrates |
| A07DA | | Antipropulsives | C01DX | Other vasodilators used for cardiac diseases |
| A07EC | | Aminosalicylic acid and similar agents | C01EA | Prostaglandins |
| A07FA | | Antidiarrheal microorganisms | C01EB | Other cardiac preparations |
| A09AA | | Enzyme preparations | C02CA | Alpha-adrenoreceptor antagonists |
| A10AB | | Insulins and analogs for injection, fast-acting | C02DB | Hydrazinophthalazine derivatives |
| A10AD | | Insulins and analogs for injection, intermediate- or long-acting combined with fast-acting | C02DD | Nitroferricyanide derivatives |
| A10AE | | Insulins and analogs for injection, long-acting | C03AA | Thiazides, plain |
| A10BA | | Biguanides | C03BA | Sulfonamides, plain |
| A10BB | | Sulfonylureas | C03CA | Sulfonamides, plain |
| A10BD | | Combinations of oral blood glucose lowering drugs | C03DA | Aldosterone antagonists |
| A10BF | | Alpha glucosidase inhibitors | C03EA | Low-ceiling diuretics and potassium-sparing agents |
| A10BG | | Thiazolidinediones | C04AC | Nicotinic acid and derivatives |
| A10BH | | Dipeptidyl peptidase 4 (DPP-4) inhibitors | C04AD | Purine derivatives |
| A10BX | | Other blood glucose lowering drugs, excluding insulins | C04AE | Ergot alkaloids |
| A11CC | | Vitamin D and analogs | C04AF | Enzymes |
| A11DA | | Vitamin D and analogs | C04AX | Other peripheral vasodilators |
| A11EA | | Vitamin B-complex, plain | C05AD | Local anesthetics |
| A11GA | | Ascorbic acid (vitamin C), plain | C05BX | Other sclerosing agents |
| A11GB | | Ascorbic acid (vitamin C), combinations | C05CA | Bioflavonoids |
| A11HA | | Other plain vitamin preparations | C05CX | Other capillary stabilizing agents |
| A12AA | | Calcium | C07AA | Beta-blocking agents, nonselective |
| A12AX | | Calcium, combinations with vitamin D and/or other drugs | C07AB | Beta-blocking agents, selective |
| A12BA | | Potassium | C07AG | Alpha and beta-blocking agents |
| A12CC | | Magnesium | C07CB | Beta blocking agents, selective, and other diuretics |

Supplementary Table 4. The Anatomical Therapeutic Chemical (ATC) level 4 classification codes for drugs included in the analysis.

| ATC code | Drug class | ATC code | Drug class |
| --- | --- | --- | --- |
| C08CA | [Dihydropyridine](https://en.wikipedia.org/wiki/Dihydropyridine) derivatives | H05BA | Calcitonin preparations |
| C08DA | [Phenylalkylamine](https://en.wikipedia.org/wiki/Phenylalkylamine) derivatives | J01AA | Tetracyclines |
| C08DB | [Benzothiazepine](https://en.wikipedia.org/wiki/Benzothiazepine) derivatives | J01BA | Amphenicols |
| C09AA | ACE inhibitors, plain | J01CA | Penicillins with extended spectrum |
| C09BA | ACE inhibitors and diuretics | J01CE | Beta-lactamase-sensitive penicillins |
| C09BB | ACE inhibitors and calcium channel blockers | J01CR | Combinations of penicillins, including beta-lactamase inhibitors |
| C09CA | Angiotensin II receptor blockers (ARBs), plain | J01DB | First-generation cephalosporins |
| C09DA | Angiotensin II receptor blockers (ARBs) and diuretics | J01DC | Second-generation cephalosporins |
| C09DB | Angiotensin II receptor blockers (ARBs) and calcium channel blockers | J01DD | Third-generation cephalosporins |
| C10AA | [HMG CoA reductase](https://en.wikipedia.org/wiki/HMG_CoA_reductase) inhibitors | J01DE | Fourth-generation cephalosporins |
| C10AB | [Fibrates](https://en.wikipedia.org/wiki/Fibrate) | J01DH | Carbapenems |
| C10AC | [Bile acid](https://en.wikipedia.org/wiki/Bile_acid) sequestrants | J01EE | Combinations of sulfonamides and trimethoprim, including derivatives |
| C10AD | Nicotinic acid and derivatives | J01FA | Macrolides |
| C10AX | Other lipid modifying agents | J01FF | Lincosamides |
| C10BA | Combinations of various lipid modifying agents | J01GB | Other aminoglycosides |
| C10BX | Lipid modifying agents in combination with other drugs | J01MA | Fluoroquinolones |
| D01AC | [Imidazole](https://en.wikipedia.org/wiki/Imidazole) and [triazole](https://en.wikipedia.org/wiki/Triazole) derivatives | J01RA | Combinations of antibacterials |
| D01AE | Other antifungals for topical use | J01XA | Glycopeptide antibacterials |
| D01BA | Antifungals for systemic use | J01XC | Steroid antibacterials |
| D02AE | [Carbamide](https://en.wikipedia.org/wiki/Carbamide) products | J01XD | Imidazole derivatives |
| D03AX | Other cicatrizants | J02AB | Imidazole derivatives |
| D06AX | Other antibiotics for topical use | J02AC | Triazole and tetrazole derivatives |
| D06BA | [Sulfonamides](https://en.wikipedia.org/wiki/Sulfonamide_(medicine)) | J04AB | Antibiotics |
| D06BB | [Antivirals](https://en.wikipedia.org/wiki/Antiviral_drug) | J04AC | Hydrazides |
| D07AA | Corticosteroids, weak (group I) | J04AK | Other drugs for the treatment of tuberculosis |
| D07AB | Corticosteroids, moderately potent (group II) | J05AB | Nucleosides and nucleotides excluding reverse transcriptase inhibitors |
| D07AC | Corticosteroids, potent (group III) | J05AF | Nucleoside and nucleotide reverse-transcriptase inhibitors |
| D07AD | Corticosteroids, very potent (group IV) | J05AH | Neuraminidase inhibitors |
| D07CC | Corticosteroids, potent, combinations with antibiotics | J05AP | Antivirals for treatment of HCV infections |
| D08AG | [Iodine](https://en.wikipedia.org/wiki/Iodine) products | J05AX | Other antivirals |
| D11AH | Agents for [dermatitis](https://en.wikipedia.org/wiki/Dermatitis), excluding [corticosteroids](https://en.wikipedia.org/wiki/Corticosteroid) | J06BB | Specific immunoglobulins |
| D11AX | Other dermatologicals | M01AB | Acetic acid derivatives and related substances |
| G01AA | [Antibiotics](https://en.wikipedia.org/wiki/Antibiotic) | M01AC | Oxicams |
| G01AF | Imidazole derivatives | M01AE | Propionic acid derivatives |
| G01AX | Other anti-infectives and antiseptics | M01AG | Fenamates |
| G02AB | [Ergot](https://en.wikipedia.org/wiki/Ergot) alkaloids | M01AH | Coxibs |
| G03BA | 3-oxoandrosten-(4) derivatives | M01AX | Other anti-inflammatory and antirheumatic agents, nonsteroids |
| G03CA | Natural and semisynthetic estrogens, plain | M02AA | Anti-inflammatory preparations, nonsteroids for topical use |
| G03CX | Other estrogens | M03AB | Choline derivatives |
| G03DA | [Pregnen](https://en.wikipedia.org/w/index.php?title=Pregnen&action=edit&redlink=1)-(4) derivatives | M03AC | Other quaternary ammonium compounds |
| G03FA | Progestogens and estrogens, fixed combinations | M03BA | Carbamic acid esters |
| G03HB | Antiandrogens and estrogens | M03BC | Ethers, chemically close to antihistamines |
| G04BD | Drugs for urinary frequency and incontinence | M03BX | Other centrally acting agents |
| G04BX | Other urologicals | M03CA | Dantrolene and derivatives |
| G04CA | [Alpha-adrenoreceptor antagonists](https://en.wikipedia.org/wiki/Alpha-adrenoreceptor_antagonists) | M04AA | Preparations inhibiting uric acid production |
| G04CB | [Testosterone](https://en.wikipedia.org/wiki/Testosterone_(medication))-[5-alpha reductase inhibitors](https://en.wikipedia.org/wiki/5-alpha_reductase_inhibitors) | M04AC | Preparations with no effect on uric acid metabolism |
| G04CX | Drugs used in benign prostatic hypertrophy | M05BA | Bisphosphonates |
| H01BA | Vasopressin and analogs | M05BB | Bisphosphonates, combinations |
| H01BB | Oxytocin and analogs | M05BX | Other drugs affecting bone structure and mineralization |
| H01CB | Somatostatin and analogs | M09AB | Enzymes |
| H02AB | [Glucocorticoids](https://en.wikipedia.org/wiki/Glucocorticoid) | M09AX | Other drugs for disorders of the musculoskeletal system |
| H02BX | Corticosteroids for systemic use, combinations | N01AB | Halogenated hydrocarbons |
| H03AA | Thyroid hormones | N01AF | Barbiturates, plain |
| H03BB | Sulphur-containing imidazole derivatives | N01AH | Opioid anesthetics |
| H03CA | Iodine therapy | N01AX | Other general anesthetics |

Supplementary Table 4. The Anatomical Therapeutic Chemical (ATC) level 4 classification codes for drugs included in the analysis.

| ATC code | Drug class | ATC code | Drug class |
| --- | --- | --- | --- |
| N02AA | Natural opium alkaloids | R03DX | Other systemic drugs for obstructive airway diseases |
| N02AB | Phenylpiperidine derivatives | R05CA | Expectorants |
| N02AE | Oripavine derivatives | R05CB | Mucolytics |
| N02AF | Morphinan derivatives | R05DA | Opium alkaloids and derivatives |
| N02AJ | Opioids in combination with nonopioid analgesics | R05DB | Other cough suppressants |
| N02AX | Other opioids | R05FA | Opium derivatives and expectorants |
| N02BA | Salicylic acid and derivatives | R06AA | Aminoalkyl ethers |
| N02BE | Anilides | R06AB | Substituted alkylamines |
| N03AA | Barbiturates and derivatives | R06AD | Phenothiazine derivatives |
| N03AE | Benzodiazepine derivatives | R06AE | Piperazine derivatives |
| N03AF | Carboxamide derivatives | R06AX | Other antihistamines for systemic use |
| N03AG | Fatty acid derivatives[edit] | R07AB | Respiratory stimulants |
| N03AX | Other antiepileptics | R07AX | Other respiratory system products |
| N04AC | Ethers of tropine or tropine derivatives | S01AA | Antibiotics |
| N04BA | Dopa and dopa derivatives | S01AD | Antivirals |
| N04BC | Dopamine agonists | S01AE | Fluoroquinolones |
| N05AA | Phenothiazines with an aliphatic side-chain | S01BA | Corticosteroids, plain |
| N05AB | Phenothiazines with a piperazine structure | S01BC | Anti-inflammatory agents, nonsteroids |
| N05AD | Butyrophenone derivatives | S01CA | Corticosteroids and anti-infectives in combination |
| N05AH | Diazepines, oxazepines, thiazepines and oxepines | S01EA | Sympathomimetics in glaucoma therapy |
| N05AX | Other antipsychotics | S01EB | Parasympathomimetics |
| N05BA | Benzodiazepine derivatives | S01EC | Carbonic anhydrase inhibitors |
| N05BB | Diphenylmethane derivatives | S01ED | Beta blocking agents |
| N05BE | Azaspirodecanedione derivatives | S01EE | Prostaglandin analogs |
| N05CD | Benzodiazepine derivatives | S01FA | Anticholinergics |
| N05CF | Benzodiazepine-related drugs | S01GX | Other antiallergics |
| N06AA | Nonselective monoamine reuptake inhibitors | S01HA | Local anesthetics |
| N06AB | Selective serotonin reuptake inhibitors | S01JA | Coloring agents |
| N06AX | Other antidepressants | S01KA | Viscoelastic substances |
| N06BX | Other psychostimulants and nootropics | S01KX | Other surgical aids |
| N06DA | Anticholinesterases | S01XA | Other ophthalmologicals |
| N06DX | Other anti-dementia drugs | S02AA | Anti-infectives |
| N07AA | Anticholinesterases | S02CA | Corticosteroids and anti-infectives in combination |
| N07AB | Choline esters | S03AA | Anti-infectives |
| N07AX | Other parasympathomimetics | V03AB | Antidotes |
| N07CA | Antivertigo preparations | V03AE | Drugs for treatment of hyperkalemia and hyperphosphatemia |
| N07XX | Other nervous system drugs | V03AF | Detoxifying agents for antineoplastic treatment |
| P01AB | Nitroimidazole derivatives | V04CA | Tests for diabetes |
| P01BA | Aminoquinolines | V04CH | Tests for renal function and ureteral injuries |
| P02BA | Quinoline derivatives and related substances | V04CX | Other diagnostic agents |
| R01AD | Corticosteroids | V06DB | Fat/carbohydrates/proteins/minerals/vitamins, combinations |
| R01AX | Other nasal preparations | V06DX | Other combinations of nutrients |
| R01BA | Sympathomimetics | V07AB | Solvents and diluting agents, including irrigating solutions |
| R02AA | Antiseptics | V08AA | Water-soluble, nephrotropic, high osmolar X-ray contrast media |
| R02AD | Anesthetics, local | V08AB | Water-soluble, nephrotropic, low osmolar X-ray contrast media |
| R03AC | Selective beta-2-adrenoreceptor agonists | V08BA | Barium sulfate containing X-ray contrast media |
| R03AK | Adrenergics in combination with corticosteroids or other drugs, excluding anticholinergics | V08CA | Paramagnetic contrast media |
| R03BA | Glucocorticoids | V08CB | Superparamagnetic contrast media |
| R03BB | Anticholinergics | V09BA | Technetium (99mTc) compounds |
| R03CA | Alpha- and beta-adrenoreceptor agonists | V09CA | Technetium (99mTc) compounds |
| R03CC | Selective beta-2-adrenoreceptor agonists | V09FX | Various thyroid diagnostic radiopharmaceuticals |
| R03CK | Adrenergics and other drugs for obstructive airway diseases | V09GA | Technetium (99mTc) compounds |
| R03DA | Xanthines | V09GX | Other cardiovascular system diagnostic radiopharmaceuticals |
| R03DC | Leukotriene receptor antagonists | V09IX | Other diagnostic radiopharmaceuticals for tumor detection |
